# Effectiveness of digital platform in reducing unintentional medication discrepancies at transition of care from hospital discharge to primary healthcare settings: a randomised controlled trial

**DOI:** 10.1186/s12875-025-02904-z

**Published:** 2025-07-02

**Authors:** Yen Yen Phang, Jew Win Kuan, Ai Ling Oh, Chuo Yew Ting, Nor Anizah Osman, Stephen Moses

**Affiliations:** 1 Faculty of Medicine and Health Sciences, Universiti Malaysia Sarawak (UNIMAS), Kota Samarahan, Malaysia; 2 Pharmacy Service Division, Sarawak State Health Department, Ministry of Health, Kuching, Malaysia; 3 Pharmacy Department, Sarawak General Hospital, Ministry of Health, Kuching, Malaysia

**Keywords:** Medication reconciliation, Medication safety, Unintentional medication discrepancies, Primary healthcare, Transition of care, Digital platform

## Abstract

**Background:**

Modifications to medication regimens during transitions of care between different healthcare settings often lead to unintentional medication discrepancies (UMDs). The MedBook Portal, a simple digital platform that enables the sharing of patient medication records among healthcare facilities under Ministry of Health, was developed to facilitate medication reconciliation at primary health clinic (PHC) after hospital discharge. This study aimed to determine the effectiveness of MedBook Portal in reducing UMDs in the first prescription during the first PHC visit after hospital discharge.

**Methods:**

This two-arm, parallel, non-blinded, randomised controlled trial was conducted at four public hospitals and ten public PHCs in Sarawak, Malaysia, from May 2023 to July 2024. Adult patients aged ≥ 18 years in general medical wards, discharged from hospitals, and referred to selected PHCs were recruited. In the Standard Care group, PHC doctors performed standard medication reconciliation by reviewing patients’ medical records on their home-based medical cards and discharge notes, if available. In the MedBook Portal group, in addition to this process, PHC doctors logged into MedBook Portal to access the discharge prescription before issuing a new prescription.

**Results:**

Among the 339 eligible subjects randomised into MedBook Portal group and Standard Care group, 307 participants (147 MedBook Portal, 160 Standard Care) were analysed after excluding those for whom the intervention was not performed (*n* = 11) and those with no prescription (*n* = 21). The incidence rate of prescription with UMDs was significantly lower in the MedBook Portal group (5/147, 3.4%) compared to the Standard Care group (30/160, 18.8%) (*p* < 0.001). The most common UMD was drug omission (54.4%). Multivariable logistic regression showed that the presence of MedBook Portal reduced the odds of UMDs by 87% (adjusted OR 0.134, 95% CI 0.049–0.336, *p* < 0.001), whereas each additional comorbidity increased the odds by 52% (adjusted OR 1.520, 95% CI 1.158–2.020, *p* = 0.003).

**Conclusions:**

Medication reconciliation using the MedBook Portal effectively reduces UMDs during the transition of care from hospital discharge to PHCs, enhancing patient safety across the continuum of care.

**Trial registration:**

ClinicalTrials.Gov (NCT06517160) was registered retrospectively on 19 September 2024.

**Supplementary Information:**

The online version contains supplementary material available at 10.1186/s12875-025-02904-z.

## Introduction

The complex processes and human interactions involved in transitions of care between different healthcare settings poses a significant risk of medication errors, especially for patients with multiple comorbidities and complex medication regimens. Medication reconciliation emerges as a cornerstone in mitigating the risks associated with medication errors across different healthcare settings.

Medication reconciliation is a formal process in which healthcare professionals partner with patients to ensure accurate and complete medication information transfer at interfaces of care [1]. The process aims to mitigate potential medication errors and enhance patient safety. According to the Joint Commission on Accreditation of Healthcare Organisations, medication reconciliation involves five essential steps: (i) develop a list of current medications; (ii) develop a list of medications to be prescribed; (iii) compare the medication on the two lists; (iv) make clinical decisions based on the comparison; and (v) communicate the new list to appropriate caregivers and to the patient [2].

During medication reconciliation, medication discrepancies (MDs) or differences in the medication regimen between two or more healthcare settings, or between what was prescribed and what was consumed by patients, may emerge. Identifying and resolving MD is essential to ensure the accuracy and appropriateness of a patient’s medication regimen. While some intentional medication discrepancies (IMDs) are justified, guided by the patient’s new clinical condition [3], and require proper communication to subsequent healthcare providers and patients, others pose a significant risk to patient safety. This unjustified variation, termed unintentional medication discrepancy (UMD), can potentially result in adverse drug events [4, 5], treatment inefficacy, or therapeutic duplication.

Alqenae et al. (2020) conducted a systematic review of 54 studies examining the prevalence and nature of medication errors following discharge from hospital to community settings [5]. The median rate of medication errors (the study defined as errors related to professional practice, health care products, procedures, and systems, including prescribing, order communication, product labelling, packaging, and nomenclature, compounding, dispensing, distribution, administration, education, monitoring, and use) and UMDs (the study defined as unexplained differences in documented medication regimens across different cite of care) after discharge were 53% [IQR 33-60.5] (*n* = 5) and 50% [IQR 39–76] (*n* = 11), respectively. Among these studies, seven reported adverse drug event rates, with a median of 19% of discharged patients affected [IQR 16–24].

Despite the recognised benefits of medication reconciliation, challenges persist in its implementation worldwide. Studies have identified several barriers, including a lack of role clarity among healthcare providers [6], poor interprofessional communication [7], and incompatibility of handwritten and electronic systems [7]. Additionally, patient-related factors such as medication non-adherence and limited patient’s health literacy further complicate the reconciliation process [7].

Numerous studies have demonstrated the incidence of prescriptions with UMDs during admission and discharge processes [8, 9]. Lehnbom et al. (2014) performed a systematic review involving 83 studies, three of which reported a higher proportion of patients with one or more UMDs at discharge compared to admission (41.0% vs. 38.0%, 71.3% vs. 33.7%, and 40% vs. 19%, respectively) [8]. This systematic review aimed to examine the evidence regarding the effectiveness of medication reconciliation and medication review in improving clinical outcomes across hospitals, community settings, and aged care facilities. Notably, the review reported that the proportion of patients with at least one MD ranged from 14.1 to 98.2% in the community and aged care settings. However, this figure reflected overall MDs rather than specifically UMD.

In a Malaysia context, Law and Chong (2017) conducted a prospective observational study on 65 patients discharged from internal medicine wards at a tertiary hospital [9]. Their findings revealed that 40% of patients experienced UMDs during the discharge process.

Studies on factors associated with MDs have primarily focused on hospital admission settings and hospital discharge [9, 10]. For example, Hias et al. (2017) conducted a systematic review of 35 cohort studies on the factors for UMDs in preadmission medication [10]. The review identified older age and a higher number of preadmission medications as the most frequently cited predictors for preadmission medication related MDs. Nine of out 24 studies reported age as a significant result. One study from the review found that patients aged ≥ 85 years had a significantly higher risk of preadmission MDs compared to those aged < 50 years (RR 0.34, 95% CI 0.16–0.73, *p* < 0.05) in a multivariable analysis. Thirteen out of 26 studies showed a significant association between the number of preadmission medication and UMDs after adjusting for confounders. The odds ratio per additional medication reported in one of the studies is 1.19 (95% CI 1.10–1.29, *p* < 0.001). In addition, Law and Chong (2017) reported that number of discharge medications was significantly associated with UMDs (adjusted OR 1.198, 95% CI 1.026–1.399, *p* = 0.022) [9].

To our knowledge, no study has investigated the factors associated with UMD in primary care settings after hospital discharge. The most relevant studies were, for example, Coletti et al. (2015), which found that the number of medications predicted the presence of MDs in a multivariate analysis (adjusted OR 1.18, 95% CI 1.10–1.27, *p* < 0.001) among 328 outpatients in a university hospital-affiliated, community-based primary care practice [11]. Al-Dahshan & Kehyayan (2021) reported that various comorbidities − including diabetes mellitus (adjusted OR 1.46; 95% CI 1.26–1.70, *p* < 0.05), hypertension (adjusted OR 1.21; 95% CI 1.02–1.42, *p* < 0.05), cardiovascular diseases (adjusted OR 1.38; 95% CI 1.18–1.60, *p* < 0.05), asthma (adjusted OR 1.21; 95% CI 1.04–1.41, *p* < 0.05), gastroesophageal reflux disease (adjusted OR 2.99; 95% CI 2.43–3.69, *p* < 0.05), and arthritis (adjusted OR 1.30; 95% CI 1.11–1.52, *p* < 0.05) − were significant predictors of potentially inappropriate medications in a cross sectional study [12]. This study analysed electronic medical records from 5,639 patients aged 65 and older who attended 23 primary healthcare centres in Qatar.

With the advancement of digital tools, Wang et al. (2017) performed a systematic review on 13 studies to evaluate the impact of electronic medication tools on UMDs at hospital admission and hospital discharge [13]. The meta-analysis demonstrated a significant reduction in the number of medications with UMDs (RR 1.85, 95% CI 1.55–2.21, *p* < 0.05). However, no statistically significant difference was observed in the number of patients with UMDs (RR 2.74, 95% CI 0.59–12.73, *p* > 0.05).

Similarly, Killin et al. (2021) conducted a systematic review examining the impact of in-hospital electronic or enhanced medication reconciliation compared to basic medication reconciliation on medication errors, MDs, and adverse drug events, primarily at the point of admission and discharge [14]. The review included ten studies (six electronic medication reconciliation and four enhanced medication reconciliation), concluded that electronic medication reconciliation reduced the odds of medication errors (adjusted OR 0.39, 95% CI 0.18–0.87, *p* = 0.021; adjusted OR 0.57, 95% CI 0.44–0.74, *p* < 0.001). Although electronic medication reconciliation tended to lower the risk of adverse drug events, the findings were limited by inconsistencies in study settings, interventions, and outcome definitions.

Ciapponi et al. (2021) conducted a systematic review of 65 studies focusing on the impact of medication reconciliation on medication errors and potential adverse drug events for adults in hospital settings, such as inpatient care units, outpatient care settings, and accident and emergency departments [15]. An analysis of two studies indicated moderate-certainty evidence supporting the effectiveness of Computerised Physician Order Entry (CPOE) or Clinical Decision Support Systems (CDSS) in reducing medication errors compared to paper-based systems (OR 0.74, 95% CI 0.31–1.79, *p* < 0.05).

Collectively, these studies demonstrate the benefits of electronic medication reconciliation in reducing MDs at hospital setting. However, there remains a notable gap in the literature regarding the impact of such digital systems on UMDs in primary care setting and during transition of care between hospital and primary care setting.

While reconciling patients’ past medications upon admission has become routine—especially in general medical wards at tertiary healthcare institutions in Malaysia—structured medication reconciliation upon and after hospital discharge remains lacking [16]. This gap potentially exposes patients to UMDs during transitions of care from hospitals to primary healthcare settings. To address such safety risks, the World Health Organisation recommends the development of tools to help patients protect themselves from medication-related harm [17]. Personal medical records are among these tools, aimed at promoting patient engagement and empowerment individuals to safely manage their own medications. This practice is implemented in Sarawak, the largest state in Malaysia, located on Borneo Island. Patients accessing public healthcare typically present hardcopy personal medical records, known as home-based medical card, during public primary health clinic (PHC) follow-ups and hospital admissions. These records offer a practical means of maintaining continuity of care, especially in settings where integrated electronic health records are lacking. However, hospital discharges information and follow-up records from secondary or tertiary facilities are not integrated into the home-based medical card routinely. Instead, patients are provided with separate hardcopy discharge notes, which are usually prepared by non-specialists or junior doctors. These notes generally include a summary of hospital stay, diagnoses, important investigation results, and discharge medications − stating drug names, dosages and frequencies. As the content of discharge note is writer-dependent, information of discharge medications might not be included. Additionally, records of secondary or tertiary clinic follow-ups are retained at the respective facilities. Consequently, patients often receive redundant medication regimens—whether identical or differing in dosage—across various levels of care. In some cases, referred healthcare facilities may continue prescribing previous medications without being aware of patient’s updated health status, ultimately compromising a holistic treatment approach. Moreover, primary healthcare providers frequently struggle to access complete medical records due to the lack of integration in Malaysia’s healthcare information system, a challenge further exacerbated when patients fail to maintain their discharge notes − either by misplacing them or forgetting to bring them during PHC follow-ups. The absence of an integrated, computerised healthcare system remains a major barrier to medication safety, particularly in ensuring continuity of care between hospitals and primary healthcare settings [7].

Hence, we conducted a trial to evaluate the effectiveness of a digital platform in the medication reconciliation process during transition of care from hospital discharge to primary healthcare setting. The digital platform in our study, named MedBook Portal, was created by Sarawak State Health Department. It enables the sharing of patient medication records among public healthcare facilities under Ministry of Health Malaysia. Thus, doctors and pharmacists in public PHCs can assess discharge prescription from public hospitals when needed. See Additional File (Sect. 1.1) for details on the MedBook Portal.

## Methods

### Study design

This is a multicentre randomised controlled trial conducted from May 2023 to July 2024. The primary objective of the trial was to compare the incidence rate of prescription with UMDs in the first prescriptions during the first PHC visit after hospital discharge in Sarawak between MedBook Portal group and Standard Care group.

The study adhered to the Consolidated Standards of Reporting Trials (CONSORT) Statement guideline and received ethical approval from the Medical Research & Ethics Committee (MREC), Ministry of Health Malaysia [NMRR-22-02409-ODX (IIR)], prior to participant recruitment. The trial was registered retrospectively in ClinicalTrials.gov (NCT06517160).

#### Sample size calculation

The sample size estimation was based on a previously reported 49% incidence rate of MDs upon discharge [9]. To detect a clinically important difference of 15% between the groups, with 80% power and a 5% significance level, a total of 166 participants per group was required using a two-tailed z-test of proportion [18]. Considering an estimated 20% loss to follow-up, the final sample size was adjusted to 398 participants to ensure sufficient statistical power.

#### Sampling population and participants eligibility

The sampling population was adult patients aged 18 years and older in Sarawak who were discharged from four public tertiary or secondary hospitals, i.e. Sarawak General Hospital, Sibu Hospital, Sarikei Hospital, and Miri Hospital, and referred to ten public PHCs located nearby these hospitals, i.e. Batu Kawa Health Clinic, Kota Sentosa Health Clinic, Kota Samarahan Health Clinic, Jalan Oya Health Clinic, Lanang Health Clinic, Sibu Jaya Health Clinic, Miri Health Clinic, Tudan Health Clinic, Sarikei Health Clinic, and Bintangor Health Clinic.

Eligible participants were those admitted to general medical wards with comorbidities related to cardiovascular, renal, respiratory, or endocrine system. Additionally, they were required to have their home-based medical cards and be referred to the designated PHCs after hospital discharge. Patients discharged during public holidays or weekends were excluded.

#### Randomisation, blinding, and intervention

A sequence of random allocation into the MedBook Portal group and the Standard Care group in 1:1 ratio was generated using an online program available at https://www.graphpad.com/quickcalcs/randomize1/. The allocation list was securely stored and accessible only to the research coordinator. During hospital admission, the hospital study pharmacist would screen for eligibility. After obtaining informed consent, the hospital study pharmacist would assign a unique identification number to each enrolled patient according to the chronological order of enrolment and collect the required study data. Subsequently, the hospital study pharmacist would inform the research coordinator on patient’s identification number and obtain the patient’s group allocation from the securely store allocation list, ensuring allocation concealment throughout the process.

At hospital discharge, each enrolled patient was provided with a hardcopy discharge note as usual and the PHC appointment date. For the MedBook Portal group, the hospital study pharmacist would attach a memo called MedBook Portal Notice at the front of the patient’s home-based medical card. See Additional File (Sect. 1.2) for details on the MedBook Portal Notice. For Standard Care group, no such memo would be attached. The hospital study pharmacist would reconcile and upload the reconciled discharge prescription into MedBook Portal. This reconciliation was carried out for both study groups but was not part of routine daily practice at the time of the study.

The research coordinator would compile and send the study data from the four hospitals to the principal investigator. The principal investigator also would send a reminding message to the enrolled patients, regardless which group they were assigned to, of the PHC appointment dates via WhatsApp or phone call.

For the Standard Care group, the principal investigator would prepare and send each participating PHC study pharmacist their respective list of enrolled Standard Care group patients along with their PHC appointment dates. When the patients attending PHC, standard medication reconciliation would be performed, of which PHC doctor would review patients’ medical records on their home-based medical cards and discharge notes, if provided by the patient. Patient would be given the first prescription, which they would then hand over to the PHC pharmacist at the PHC pharmacy to collect their medications. PHC pharmacist would compare this prescription against the patients’ home-based medical cards and discharge notes, if available. Any identified MDs would be confirmed with the PHC doctor. For the purpose of this study, the PHC study pharmacist would check the given list and access MedBook Portal, usually one to five days after the PHC visit. Any identified MDs between discharge and current prescription would be confirmed with the PHC doctor. PHC study pharmacist would call patient to come back if any significant medication change was needed. Subsequently, the PHC study pharmacist would enter and submit the data to the principal investigator.

**Fig. 1:**
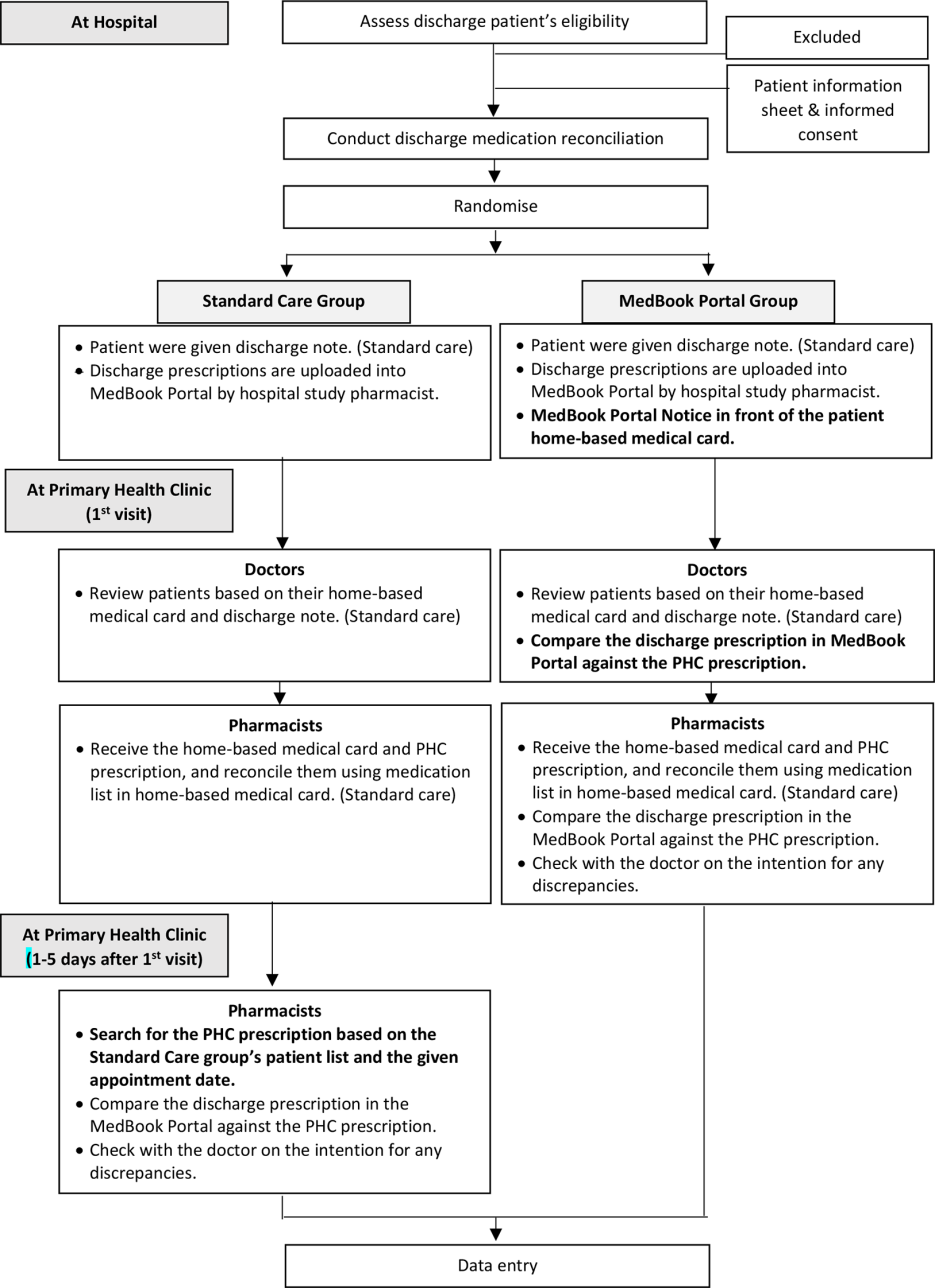
: Study flow chart

For the MedBook Portal group, on top of the same process that would occur when the patients attended PHC, PHC doctor would notice the MedBook Portal Notice attached at the front of the patient’s home-based medical card and log into the MedBook Portal to access the discharge prescription before issuing a prescription. Patient would be given the first prescription, which they would then hand over to the PHC pharmacist at the PHC pharmacy to collect their medications. Upon receiving the prescription from the patient and noticing the MedBook Portal Notice, PHC study pharmacist would also access the MedBook Portal. Any identified MDs between discharge and current prescription were confirmed with the PHC doctor at the same PHC visit setting. After completion of PHC visit and intervention process, PHC study pharmacist would enter and submit the data to the principal investigator. The study flow chart is illustrated in Fig. 1.

The research coordinator communicated with the hospital study pharmacists and principal investigator only and had no direct contact with patients, PHC study pharmacists or PHC doctors. Principle investigator communicated with the research coordinator, patients, hospital study pharmacists and PHC study pharmacists but not with PHC doctors, and performed analysis based on the data received. Due to the nature of the intervention, blinding was not feasible for patients, hospital study pharmacists, PHC doctors, or PHC study pharmacists.

#### Outcomes assessment

MD is defined as any variation or difference in medication regimen identified when comparing an old medication list with newly prescribed medications. IMD is a justified MD due to a change in the patient’s clinical condition. UMD is an unjustified MD identified after checking with prescriber and it is a medication error, specifically prescribing error. MDs are classified into discrepant dose, discrepant frequency, therapeutic duplication, drug omission, drug addition, and others (Table 1), of which the definition was modified from Kennelty et al. (2016) and Wong et al. (2008) [19, 20]. See Additional File (Figure F) for an illustration of the MDs detected during medication reconciliation at hospital discharge and the first PHC visit.

**Table 1:** Type of MD identified between the first prescription at first PHC visit and the discharge prescription

Type of MD	Description	Example
Discrepant dose	A difference in medication dosage between the first PHC and discharge prescriptions.	A patient has been discharged with Losartan 100 mg. However, the first PHC prescription is Losartan 50 mg.
Discrepant frequency	A difference in medication frequency between the first PHC and discharge prescriptions.	A patient has been discharged with Frusemide 40 mg BD. However, Frusemide 40 mg OD is prescribed in the first PHC prescription.
Therapeutic Duplication	Multiple agents from the same therapeutic class.	Mefenamic acid and Diclofenac are prescribed concurrently.
Drug omission	Medication is not prescribed in the first PHC prescription but is listed in the discharge prescription.	A patient was discharged with Metoprolol, Losartan, and Metformin. However, only Metoprolol and Metformin are prescribed in the first PHC prescription.
Drug addition	Medication is prescribed in the first PHC prescription but not listed in the discharge prescription.	On the first PHC visit, the patient is prescribed with Metoprolol, Losartan and Metformin. However, only Metoprolol and Metformin are listed in the discharge prescription.
Others	Any MDs that do not fit into any of the previously mentioned categories, such as a different formulation of the same item is prescribed.	Example 1: A patient has been discharged with Budesonide inhaler. However, Budesonide nasal spray is prescribed in the first PHC prescription. Example 2: A patient has been discharged with Gliclazide 120 mg MR OD. However, Gliclazide 120 mg OD is prescribed in the first PHC prescription.

BD: Twice Daily; MD: Medication Discrepancy (see text for definition); MR: Modified-release; OD: Once Daily; PHC: Public Health Clinic

#### Endpoints

The primary endpoint of the study was to compare the incidence rate of prescriptions with UMDs in the first prescriptions during the first PHC visit after hospital discharge between MedBook Portal group and Standard Care group.

The secondary endpoints were the type of UMDs and factors associated with the UMDs.

### Statistical analysis

The analysis was conducted using a per-protocol approach. All data were analysed using jamovi (2022) (Version 2.3). Descriptive statistics, including frequency and percentage for categorical variables, and mean and standard deviation for continuous variables, were used to describe the socio-demographic characteristics of the participants.

Categorical variables were reported in frequencies and percentages and compared using the Chi-square test. Continuous variables were assessed for normality using the One-Sample Kolmogorov-Smirnov Test. For normally distributed data, comparisons were made using independent t-tests, while the Mann-Whitney U test was employed for non-normally distributed data. Normally distributed variables were reported as mean (standard deviation, SD), while non-normally distributed variables were reported as median (interquartile range, IQR).

Factors associated with UMDs were first analysed using univariate logistic regression. The variables included presence of intervention, age, number of comorbidities, number of medications, type of medications, and category of prescriber. Variables with a p-value < 0.1 in the univariate analysis were included in the multivariable logistic regression model. All statistical tests were two-tailed, and *p* < 0.05 was considered statistically significant.

## Result

**Fig. 2:**
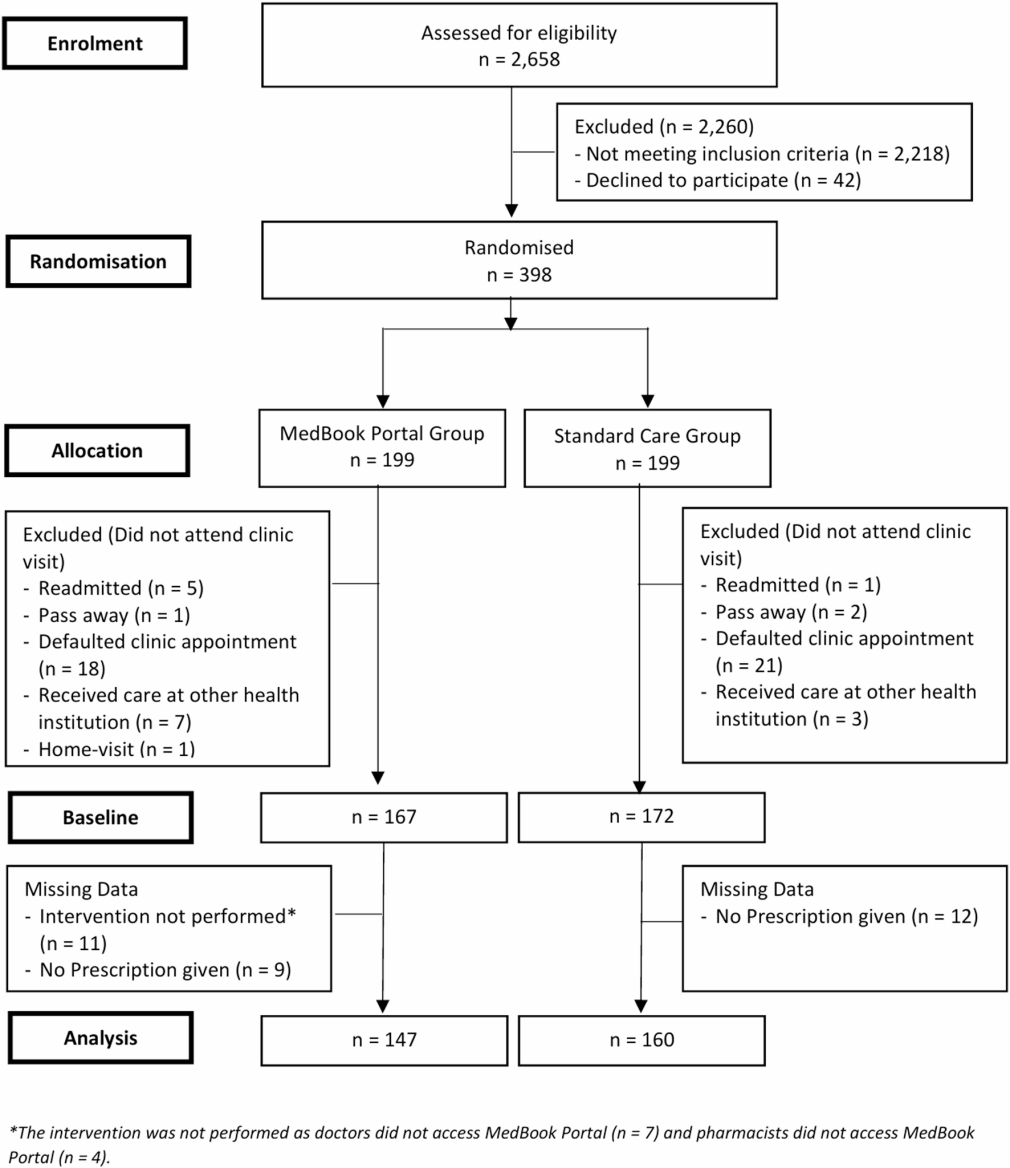
Flow chart of the respondents through the trial

Of the 2,658 screened patients, 440 (16.6%) met the inclusion criteria. However, 42 patients refused to participate, resulting in an initial cohort of 398 participants, who were randomly assigned to the MedBook Portal group (*n* = 199) and the Standard Care group (*n* = 199) (Fig. 2). Out of a total of 339 participants who attended the PHC, 147 and 160 participants in the MedBook Portal and Standard Care groups, respectively, were included in the analysis after excluding those for whom the intervention was not performed (*n* = 11) and those with no prescription (*n* = 21).

The baseline socio-demographic characteristics of the 307 participants included for analysis are presented in Table 2. The baseline characteristics of the initial cohort of 398 participants are provided in the Additional File (Table A). Among the 307 participants, majority were female (51.8%) and 45.3% were Sarawak Native. The mean age of the participants was 63 years (SD 13.7). The median number of comorbidities among the participants was 3 (IQR 2–4). On discharge, participants were prescribed a mean of 6.8 medications (SD 2.8), and during the first PHC visit, the mean number of medications prescribed was 6.0 (SD 2.9). Almost all (99%) of the prescriptions during the first PHC visit were prepared by Medical Officer. There was no significant difference between the groups, except that the MedBook Portal group had a higher number of medications in the discharge prescription compared to the Standard Care group (mean 7.3 vs. 6.3, respectively; *p* = 0.001) and a higher number of medications in the first PHC prescription (mean 6.6 vs. 5.6, respectively; *p* = 0.002).

**Table 2:** Comparison of baseline characteristics between the groups included in analysis (*n* = 307)

Variables	Overall (*n* = 307)	MedBook Portal Group (*n* = 147)	Standard Care Group (*n* = 160)	*p* value
Study sites, n (%)				
Sarawak General Hospital	136 (44.3)	63 (42.9)	73 (45.6)	0.799^a^
Sarikei Hospital	81 (26.4)	37 (25.2)	44 (27.5)	
Sibu Hospital	74 (24.1)	39 (26.5)	35 (21.9)	
Miri Hospital	16 (5.2)	8 (5.4)	8 (5.0)	
Age, mean years (SD)	63.3 (± 13.7)	64.0 (± 13.3)	62.8 (± 14.1)	0.456^b^
Gender, n (%)				
Female	159 (51.8)	76 (51.7)	83 (51.9)	0.976^a^
Male	148 (48.2)	71 (48.3)	77 (48.1)	
Ethnicity, n (%)				
Sarawak Native	139 (45.3)	74 (50.4)	65 (40.6)	0.238^a^
Chinese	101 (32.9)	44 (29.9)	57 (35.6)	
Malay	66 (21.5)	28 (19.0)	38 (23.8)	
Indian	1 (0.3)	1 (0.7)	0 (0.0)	
Number of comorbidities, median (IQR)	3 (2–4)	3 (3–4)	3 (2–4)	0.184^c^
Number of medications in Discharge Prescription, mean (SD)	6.76 (± 2.75)	7.29 (± 2.71)	6.27 (± 2.71)	**0.001** ^**b**^
Number of medications in First Prescription, mean (SD)	6.04 (± 2.88)	6.56 (± 2.85)	5.56 (± 2.83)	**0.002** ^**b**^
Prescriber category of First Prescription, n (%)				
Medical Officer	305 (99.3)	146 (99.3)	159 (99.4)	0.952^a^
Family Physician Specialist	2 (0.7)	1 (0.7)	1 (0.6)	

^a^Pearson’s chi-squared test

^b^Independent t test

^c^Mann-Whitney U test

IQR: Interquartile Range; SD: Standard Deviation

Out of the 307 prescriptions included for analysis (one prescription per patient), 229 (74.6%) and 35 (11.4%) prescriptions had at least one MD and UMD, respectively (Table 3). On average, each prescription contained 2.2 MDs (SD 2.2) and 0.2 UMDs (SD 0.8).

Comparatively, the MedBook Portal group (*n* = 147) had a lower incidence rate of prescriptions with MDs than the Standard Care group (*n* = 160), with 67% (*n* = 98) versus 82% (*n* = 131), respectively (*p* = 0.002). Additionally, the MedBook Portal group had fewer MDs per prescription, averaging 1.8 (SD 1.9) compared to 2.7 (SD 2.4) in the Standard Care group (*p* < 0.01).

**Table 3:** Comparison of MDs between the groups in the first prescriptions during the first PHC visit after hospital discharge (*n* = 307)

Variables	Overall (*n* = 307)	MedBook Portal Group (*n* = 147)	Standard Care Group (*n* = 160)	*p* value
Number of prescriptions with MDs, n (%)				
Yes	229 (74.6)	98 (66.7)	131 (81.9)	**0.002** ^**a**^
No	78 (25.4)	49 (33.3)	29 (18.1)	
Number of prescriptions with UMDs, n (%)				
Yes	35 (11.4)	5 (3.4)	30 (18.8)	**< 0.001** ^**a**^
No	272 (88.6)	142 (96.6)	130 (81.2)	
Number of MDs per prescription, mean (SD)	2.24 (± 2.23)	1.76 (± 1.91)	2.69 (± 2.41)	**< 0.001** ^**b**^
Number of UMDs per prescription, mean (SD)	0.22 (± 0.81)	0.03 (± 0.18)	0.39 (± 1.08)	**< 0.001** ^**b**^

^a^Pearson’s chi-squared test

^b^Mann-Whitney U test

MD: Medication Discrepancy (see text for definition); PHC: Public Health Clinic; SD: Standard Deviation; UMD: Unintentional Medication Discrepancy (see text for definition)

A total of 692 MDs were identified across 307 prescriptions during the first PHC visit after hospital discharge (Table 4). Drug addition was the most common MD (44.5%), followed by drug omission (40.5%), discrepant dose (12.3%), discrepant frequency (2.3%), and others (0.4%).

**Table 4:** Type of MD in the first prescription during the first PHC visit after hospital discharge (*n* = 692)

Types of MD	Number of MD / Total MD (%)		
	MD (*n* = 692)	UMD (*n* = 68)	IMD (*n* = 624)
Drug Addition	308 (44.5)	15 (22.0)	293 (47.0)
Drug Omission	280 (40.5)	37 (54.4)	243 (38.9)
Discrepant Doses	85 (12.3)	8 (11.8)	77 (12.3)
Discrepant Frequency	16 (2.3)	7 (10.3)	9 (1.5)
Others	3 (0.4)	1 (1.5)	2 (0.3)
Therapeutic Duplication	0 (0.0)	0 (0.0)	0 (0.0)

IMD: Intentional Medication Discrepancy (see text for definition); MD: Medication Discrepancy (see text for definition); PHC: Public Health Clinic; UMD: Unintentional Medication Discrepancy (see text for definition)

### Primary endpoint

The incidence rate of prescription with UMDs in the first prescriptions during the first PHC visit after hospital discharge in the MedBook Portal group (*n* = 147) was significantly lower than in the Standard Care group (*n* = 160), 5 (3.4%) versus 30 (18.8%) (*p* < 0.001) (Table 3). On average, the number of UMDs per prescription was 0.03 in the MedBook Portal group, lower than 0.39 in the Standard Care group (*p* < 0.001).

### Secondary endpoints

A total of 68 UMDs were identified across 35 prescriptions during the first PHC visit after hospital discharge (Table 4). Drug omission was the most common UMD (54.4%), followed by drug addition (22.0%), discrepant dose (11.8%), discrepant frequency (10.3%), and others (1.5%).

Three out of six factors – presence of intervention i.e. MedBook Portal, patient age, and number of comorbidities showed a significant association with UMDs in the univariate logistic regression analysis (Table 5). In contrast, the remaining predictors–number of medications, category of prescriber, and type of medications − demonstrated no statistically significant association. Notably, category of prescriber and type of medications yielded non-informative results, with extremely wide confidence intervals (0.000 to infinity) and non-significant p-values (*p* > 0.05). Due to the lack of statistical interpretability, these variables were not presented in the Table 5. However, multivariable logistic regression analysis revealed that only the presence of intervention (adjusted OR 0.134, 95% CI 0.049–0.336, *p* < 0.001) and number of comorbidities (adjusted OR 1.520, 95% CI 1.158–2.020, *p* = 0.003) remained significant in the model. The intervention acted as a protective factor − reducing the odds of UMD by 87%−whereas each additional comorbidity was a risk factor, increasing the odds of UMD by 52%.

**Table 5:** Logistic regression analysis of factors for UMDs in the first prescription during the first PHC visit after hospital discharge

Factors	UMDs			
	Crude OR (95% CI)	*p* value	Adjusted OR (95% CI)	*p* value
Presence of intervention	**0.153** **(0.058–0.374)**	**< 0.001**	**0.134** **(0.044–0.336)**	**< 0.001**
Age	**1.023** **(0.997–1.052)**	**0.096**	1.014 (0.985–1.044)	0.343
Number of comorbidities	**1.495** **(0.165–1.933)**	**0.002**	**1.520** **(1.158–2.020)**	**0.003**
Number of medications	1.030 (0.911–1.162)	0.629	-	-

CI: Confidence Interval; OR: Odds Ratio; PHC: Public Health Clinic; UMD: Unintentional Medication Discrepancy (see text for definition)

## Discussion

### UMDs in the first prescription during the first PHC visit after hospital discharge

The utilisation of the MedBook Portal resulted in a lower incidence rate of prescriptions with UMDs in the first prescription during the first PHC visit after hospital discharge when compared to Standard Care, 3.4% versus 18.8%, i.e. 15.4% difference. The findings of this study align with previous research demonstrating the efficacy of interventions involving medication reconciliation with the support of electronic health records [21–23]. Tamblyn et al. (2019) conducted a cluster randomised trial involving 3,491 patients discharged from an academic hospital to community settings to evaluate UMD. The study reported that UMDs were significantly reduced in the intervention group compared with the control group, 26.4% versus 56.0%, i.e. 29.6% difference [23]. Similarly, Garcia-Molina Saez et al. (2016) found that UMDs upon discharge were reduced from 42.2 to 19.8%, i.e. 22.4% difference, during the intervention period of a computerised pharmaceutical system in a quasi-experimental interrupted time series study [22]. Allison et al. (2015) observed a reduction in antibiotics-related medication errors from 30 to 15% after implementing an electronic discharge medication reconciliation tool system in a retrospective pre-post interventional study of 100 subjects in an academic hospital [21].

The incidence rate of prescription with UMDs in the first prescriptions during the first PHC visit after hospital discharge was high in the Standard Care group, 18.8%. Despite this, the incidence rate of prescription with UMDs in our study was lower compared to findings by Tamblyn et al. (2019). This could be explained by the use of home-based medical cards, which are routinely carried by patients in our setting. In addition, PHC doctors would review patients’ discharge notes if provided by the patient, which might contain information on discharge medications and serve as an important reference during medication reconciliation. Furthermore, PHC doctors may have been more cautious in prescribing due to awareness of being observed in the study. Tamblyn et al. (2019) reported a 56% incidence rate of prescription with UMDs in standard care group in a clustered randomised trial [24]. They suggested this may have been overestimated, as their study relied on health record documentation, which may be incomplete, to determine if there was a rationale for omissions, dosage changes, and therapy duplications. Our study had direct access to real-time records, minimising the likelihood of assuming UMDs.

In this study, drug omission was the most prevalent type of UMD (54.4%) in the first prescription during the first PHC visit after hospital discharge. This result was consistent with the findings by Aires-Moreno et al. (2021) who reported that drug omission (54.1%) was the most common UMD during post-hospitalisation follow-up of 248 children at paediatric clinic of the four teaching hospitals [25]. The high incidence of drug omission in this study may be attributed to the complexity of the patients’ medication regimens, with an average of 6 medications per prescription.

### Implementation of digital platform for medication records

Although Hospital Information System (HIS) with CPOE and CDSS have proven effective in reducing UMDs [26], many resource-limited settings lack access to these advanced technologies. Our study has shown that a simpler electronic system capturing the discharge prescription, like our MedBook Portal, reduces the incidence rate of prescription with UMDs in the first prescriptions during the first PHC visit after hospital discharge. See Additional File (Figure C) for the example of discharge prescription in MedBook Portal. This option should be considered in settings with limited resources.

One notable issue observed in the MedBook Portal group was the inability to conduct medication reconciliation due to internet downtime, preventing pharmacists and doctors from accessing the MedBook Portal and retrieving discharge prescription. Technical issue with the system is inevitable, highlighting the importance of a responsive technical support service to address technical issue promptly. In addition to technical support, field support for hardware is critical. Organisational support is crucial for digital platforms implementation, including training [27] and overcoming technical infrastructure challenges such as electrical power, internet connectivity, and mobile phone access, which can hinder effective utilisation of digital platforms. Providing patient information in real-time enhances communication, coordination, and productivity within healthcare regions, facilitating empowerment and multidisciplinary teamwork.

Beside implementing digital platform like MedBook Portal, multifaceted approach at the system, provider, and patient level is necessary to reduce UMDs. Various barriers hinder medication reconciliation at PHCs, particularly for patients discharged from hospitals [28]. These include limited access to medication records from external institutions [29], high patient volumes [29, 30], and time constraints [30, 31]. To address these challenges, continuous professional education programs on medication reconciliation workflows are essential to reinforce its importance among healthcare providers. Additionally, improving patients’ health literacy and understanding of their medications empower them to take an active role in their treatment, enhancing adherence and reducing errors.

The findings highlight the effectiveness of digital platform in reducing UMDs, emphasising the need for further research on their impact on long-term clinical outcomes and cost-effectiveness. Future studies should explore their broader effect on patient-level outcomes, such as glycaemic and blood pressure control, while accounting for medication adherence as baseline variable. Additionally, research should assess the cost-effectiveness of digital platform in medication reconciliation, specifically focusing on its role in mitigating UMDs throughout the phases of hospital admission, discharge, and subsequent primary healthcare monitoring.

### Factors associated with UMDs in the first prescription during the first PHC visit after hospital discharge

The study identified that the presence of intervention i.e. MedBook Portal and the number of comorbidities were significant factors of UMDs in the first prescriptions during the first PHC after hospital discharge. Conversely, patient age was found to be insignificant, aligning with the findings from previous studies by Coletti et al. (2015) and Al-Dahshan & Kehyayan (2021) examining factors affecting MDs at primary care settings [11, 12]. Coletti et al. (2015) reported that number of medications predicted the presence of MDs in a multivariate analysis (adjusted OR 1.18, 95% CI 1.10–1.27, *p* < 0.001) conducted in a university hospital-affiliated, community-based primary care practice [11]. Therefore, we conducted a multicollinearity test and found no significant collinearity between the number of comorbidities and the number of medications, or between the number of comorbidities and patient age. This confirms that the non-significant associations observed between the number of medications and UMDs, and between age and UMDs, are not due to multicollinearity.

Al-Dahshan & Kehyayan (2021) also found that comorbidities − including diabetes mellitus, hypertension, cardiovascular diseases, asthma, gastroesophageal reflux disease, and arthritis − were significant predictors of potentially inappropriate medications in a cross sectional study conducted at primary care settings [12]. This supports our observation that patients with more comorbidities tend to experience more frequent medication changes, thereby increasing the risk of UMD. The complexity of these treatment regimens, coupled with potential miscommunication between healthcare providers, increases the likelihood of discrepancies during transitions of care.

Logistic regression was employed to adjust for potential confounders and identify factors associated with UMDs. By including the intervention i.e. MedBook Portal as an independent variable along with patient age and number of comorbidities, both univariate (crude OR 0.153, 95% CI 0.058–0.374, *p* < 0.001) and multivariable (adjusted OR 0.134, 95% CI 0.044–0.336, *p* < 0.001) logistic regression analyses demonstrated that the use of the MedBook Portal significantly reduced UMDs. This finding reinforces the robustness of our findings.

### Limitation

The findings of this study should be interpreted with caution due to potential biases introduced by the study design. Firstly, the PHC study pharmacists were unblinded, leading to a potential observer bias. Secondly, recall bias may be present as the PHC study pharmacists might not promptly retrieve the first prescription for the Standard Care group on the appointment date. Consequently, the PHC study pharmacists can only check with the prescriber, usually one to five days after the PHC visit, relying on the prescriber’s memory to accurately recall the details. Thirdly, in current practice, PHC doctors can access patients’ discharge notes if the patients present the document during the visit. Thus, it was assumed that PHC doctors review all the patients recruited for the study through their usual work process.

Patients discharged during weekends or public holidays were excluded due to the unavailability of hospital study pharmacists to conduct medication reconciliation and update the MedBook Portal. This may, however, limit the generalisability of findings to usual hospital setting and in settings with limited pharmacist coverage.

The analysis was conducted using a per-protocol approach. A sensitivity analysis was performed using mode imputation for missing data. For per-protocol analysis, the relative risk of UMDs in MedBook Portal group was 0.18 (95% CI 0.07–0.46, *p* < 0.001) as compared to Standard Care group. For intention-to-treat analysis, the relative risk of UMDs in MedBook Portal group was 0.17 (95% CI 0.07–0.43, *p* < 0.001) as compared to Standard Care group, which is similar to per-protocol analysis. Thus, the missing data did not alter the finding of the study i.e. MedBook Portal was effective in reducing UMDs.

This study had a 22.9% dropout rate, which could affect reliability. However, sensitivity analyses, including intention-to-treat analysis and Best-Case/Worst-Case Scenario analyses, confirmed that the primary outcome remained consistent, supporting the study’s validity.

A possible factor contributing to the 9.8% non-attendance at clinic appointment in this study could be the provision of ample medication upon hospital discharge, which may have reduced the urgency for participants to attend their clinic appointments at the PHC. For example, some participants may have been prescribed a two-month supply of medication at discharge. Given that clinic appointments at PHCs typically occur within one week after discharge, participants might not perceive an immediate need for a new prescription, potentially leading to non-attendance.

## Conclusion

Medication reconciliation using digital platform, such as MedBook Portal, reduced UMDs in the first prescription during the first PHC visit after hospital discharge. Healthcare providers should pay particular attention to patients with multiple comorbidities, as these are prone to UMDs.

## Electronic supplementary material

Below is the link to the electronic supplementary material.

Supplementary Material 1

Supplementary Material 2

## Data Availability

Data are available upon reasonable request from the corresponding author.

## Abbreviations

BD: Twice Daily
CDSS: Clinical Decision Support Systems
CI: Confidence Interval
CONSORT: Consolidated Standards of Reporting Trials
CPOE: Computerised Physician Order Entry
HIS: Hospital Information System
IMD: Intentional Medication Discrepancy
IQR: Interquartile Range
MD: Medication Discrepancy
MR: Modified- release
MREC: Medical Research & Ethics Committee
OD: Once Daily
OR: Odds Ratio
PHC: Primary Health Clinic
RR: Relative Risk
SD: Standard Deviation
UMD: Unintentional Medication Discrepancy
