# Supplementary material for: Effectiveness of digital platform in reducing unintentional medication discrepancies at transition of care from hospital discharge to primary healthcare settings: a randomised controlled trial
Source: BMC Prim Care. 2025 Jul 2;26:206. doi: 10.1186/s12875-025-02904-z (PMC12219133; doi:10.1186/s12875-025-02904-z)
Supplement: Supplementary file 1 — Supplementary Material 1 [file 12875_2025_2904_MOESM1_ESM.pdf]

STUDY PROTOCOL

RESEARCH TITLE

A Randomised Controlled Trial to Compare MedBook Portal and Standard Care in Medication Reconciliation at Transitions of Care from Hospital Discharge to Primary Healthcare Settings

TABLE OF CONTENTS

	Page
**RESEARCH TITLE**	**1**
**TABLE OF CONTENTS**	**2**
**LIST OF TABLES**	**5**
**LIST OF FIGURES**	**6**
**LIST OF ABBREVIATIONS**	**7**
**CHAPTER 1 INTRODUCTION**	**8**
1.1 Study Background	8
1.2 Problem Statement	8
1.3 Research Question	10
1.4 Objectives	10
1.5 Study Hypotheses	11
1.6 Conceptual Framework	14
1.7 Significance of Research	15
1.8 Chapter Summary	16
**CHAPTER 2 METHODOLOGY**	**17**
2.1 Study Design	17
2.2 Operational Definition of Terms	17
2.2.1 Medication Discrepancies	17

2.2.2	Intentional Medication Discrepancy	17
2.2.3	Unintentional Medication Discrepancy	17
2.2.4	Types of Medication Discrepancies	18
2.3	Study Population	24
2.4	Study Sample and Locations	24
2.4.1	Inclusion criteria	25
2.4.2	Exclusion criteria	25
2.4.3	Withdrawal criteria	25
2.5	Sample Size Calculation	26
2.6	Study Duration	27
2.7	Data and Instruments	28
2.7.1	Data Collection Form	28
2.7.2	MedBook Portal	28
2.7.3	MedBook Portal Notice	30
2.8	Recruitment	31
2.9	Medication Reconciliation Upon Hospital Discharge	31
2.10	Randomisation and blinding	31
2.11	Intervention Group	32
2.12	Control Group	33
2.13	Data Collection	33
2.14	Outcomes	37

2.15 Data Analysis 38

2.16 Chapter Summary 39

CHAPTER 3 GANTT CHART 40

3.1 Gantt Chart 40

REFERENCES 42

APPENDICES 44

LIST OF TABLES

		Page
Table 1.1:	Study Hypotheses	12
**Table 2.1**:	Description And Example Of Each Type Of Medication Discrepancy Identified During Medication Reconciliation At Hospital Discharge	19
**Table 2.2**:	Description And Example Of Each Type Of Medication Discrepancy Identified During Medication Reconciliation At The First Follow-Up At Health Clinics After Discharge From The Hospital	21
**Table 2.3:**	Public Major Hospitals and Health Clinics Involved in this Study	24
Table 2.4:	Sample Size Allocation Of Each Hospitals	27
Table 2.5:	Schedule of Enrolment, Interventions and Assessments	35
Table 3.1:	Gantt Chart	41

LIST OF FIGURES

		Page
Figure 1.1:	Conceptual Framework	14
Figure 2.1:	Example of medication discrepancies detected during medication reconciliation	23
Figure 2.2:	MedBook Portal in smartphone version	29
Figure 2.3:	MedBook Portal in computer version	30
Figure 2.4:	MedBook Portal Notice	30
Figure 2.5:	Study Flow Chart	36

LIST OF ABBREVIATIONS

BD	Twice Daily
HC	Health Clinic
MR	Modified Release
PRN	When necessary
OD	Once Daily
TDS	Three times a day

CHAPTER 1

INTRODUCTION

1.1 Study Background

The complex processes and human interactions involved in transitions of care between different healthcare settings harbour a great risk of medication errors, particularly among patients with multiple comorbidities and complex medication regimens. Medication reconciliation is thus one of the key strategies to improve medication safety across transitions of care. It is the formal process of creating the accurate and complete list of medications through the partnership between healthcare providers and patients at all interfaces of cares to reduce potential medication errors and patient harm (1).

The four steps involved in medication reconciliation are: (1) verification: obtain the current medication list; (2) clarification: check the medication dosages; (3) reconciliation: compare and document newly prescribed and previous medications and (4) transmission: communicate the updated and verified medication list to the next healthcare provider (1). These processes involve multiple stakeholders (doctors, nurses, pharmacists, and patients) and all parties are interdependent and accounted to improve transitional care medication safety.

1.2 Problem Statement

Numerous studies showed that medication discrepancies and errors are prevalent during admission and discharge (2, 3). Reconciliation of patient's past medications upon admission has been an established routine for more than a decade among clinical pharmacists, especially in the general medical wards at tertiary healthcare institutions in Malaysia. Nonetheless, medication reconciliation upon and after hospital discharge has not been implemented in a

structured process, potentially putting patients at risk of medication errors during transitions of care from hospital to the primary healthcare setting.

Very often, local healthcare providers find it difficult to retrieve previous medical records as the healthcare information system has not been integrated across the healthcare facilities in Malaysia. This problem gets worse when patients do not compile and keep their discharge summary properly when they are discharged and followed up at the primary health clinics. Tracing the record is either involving a lengthy process or to no avail due to vague information provided by the patient or caregiver. The referred facility could have continued with the previous medications without knowing the patient's latest disease state. Thus, scattered sources of medication information among different levels of care and lack of integrated computerised healthcare information system pose a great challenge to ensure medication safety. Besides the above barriers, some other potential predictive factors associated with medication errors were patient characteristics, clinical conditions, number of medications, specific pharmacological agents, length of hospital stay and inexperienced prescribers (4).

To the best of our knowledge, there has been no study conducted in Malaysia, that aims to reduce the medication errors when patients were discharged from a tertiary or secondary hospital to primary health clinic. Past studies conducted in western countries provided limited generalizable evidence on the efficacy of their interventions in dealing with the problem in local context due to different healthcare delivery system and practices (5, 6). In addition, relevant studies conducted in Malaysia merely explore the prevalence and factors contributing to the problem and the perception of stakeholders on the feasibility of implementing medication reconciliation program upon hospital discharge (7, 8). Hence, this warrants the development of an effective measure to overcome the problem. Apart from exploring the prevalence and predictors of medication discrepancies upon discharge, an innovative intervention that aims to

reduce the medication discrepancies upon transition of care will also be introduced and implemented at the major divisions in Sarawak as our first move.

1.3 Research Question

The research question of the current study is “is MedBook Portal effective in reducing medication discrepancies upon transition of care from hospitals to primary health clinics?”.

1.4 Objectives

In general, this study aims to assess the prevalence of medication discrepancies in the first prescription upon transition of care from hospitals to primary health clinics, comparing MedBook Portal to standard care.

In specific, this study aimed:

i. To assess the prevalence of medication discrepancies in the first prescription upon transition of care from hospitals to primary health clinics, comparing MedBook Portal to standard care.
ii. To determine the prevalence of the type of medication discrepancies in the first prescription upon transition of care from hospitals to primary health clinics.
iii. To determine the prevalence of the type of medication discrepancies in discharge prescription.
iv. To identify the predictors associated with medication discrepancies in the first prescription upon transition of care from hospitals to primary health clinics.
v. To identify the predictors associated with medication discrepancies in discharge prescription.

1.5 Study Hypotheses

The study hypotheses are formulated based on the study specific objectives. For the first specific objective, the hypothesis is about the impact of MedBook Portal on the prevalence of medication discrepancies in the first prescription upon transition of care from hospitals to primary health clinics.

In terms of the second and third objectives, the hypotheses are based on the previous study conducted the hypotheses are based on the previous study conducted by Law and Chong (2017) in Malaysia. This study reported 49% of the discharge prescription were found to have a least one medication discrepancies (8).

For the fourth and fifth specific objectives, there are five variables are included as the secondary outcome of the study, namely age of patient, number of comorbidities, number of medications, type of medication (ATC categorization), and experience of prescriber (category of prescriber). All the five secondary outcomes are hypothesized to be the predictors of medication discrepancies. The hypotheses are tabulated in Table 1.1.

Table 1.1: Study Hypotheses

Specific objective 1: To assess the prevalence of medication discrepancies in the first prescription upon transition of care from hospitals to primary health clinics, comparing MedBook Portal to standard care.	
H1a	There will be a significant difference of medication discrepancies rates between the intervention group and the control group after the intervention. The estimated difference of the medication discrepancies rates is 15%, with intervention group having lower medication discrepancies rates than control group.
Specific objective 2: To determine the prevalence of the type of medication discrepancies in the first prescription upon transition of care from hospitals to primary health clinics.	
H2a	The prevalence of each type of medication discrepancy ranges from 5 – 60%, with Drug omission as the most common medication discrepancy in the first prescription upon transition of care from hospitals to primary health clinics.
Specific objective 3: To determine the prevalence of the type of medication discrepancies in discharge prescription.	
H3a	The prevalence of each type of medication discrepancy ranges from 5 – 60%, with Drug omission as the most common medication discrepancy in discharge prescription.
Specific objective 4: To identify the predictors associated with medication discrepancies in the first prescription upon transition of care from hospitals to primary health clinics.	
H4a	There is a significant association between age of patient, number of comorbidities, number of medications, type of medication (ATC categorization), experienced of prescriber (category of prescriber) and medication discrepancy in the first prescription upon transition of care from hospitals to primary health clinics.

continued

Specific objective 5: To identify the predictors associated with medication discrepancies in discharge prescriptions.	
H5a	There is a significant association between age of patient, number of comorbidities, number of medications, type of medication (ATC categorization), experience of prescriber (category of prescriber) and medication discrepancy in discharge prescription.

1.6 Conceptual Framework

The conceptual framework of the study is based on the rationale and design of the "Multicenter Medication Reconciliation Quality Improvement Study" (MARQUIS) (9). Bidirectional medication information transfer across settings via electronic health records shall ease the medication reconciliation process. Subsequently, a reduction in medication discrepancy will be the outcome of the study. In addition, predictors correlated with medication discrepancies might impact the prevalence of medication discrepancies. Figure 1.1 depicts the conceptual framework of the study.

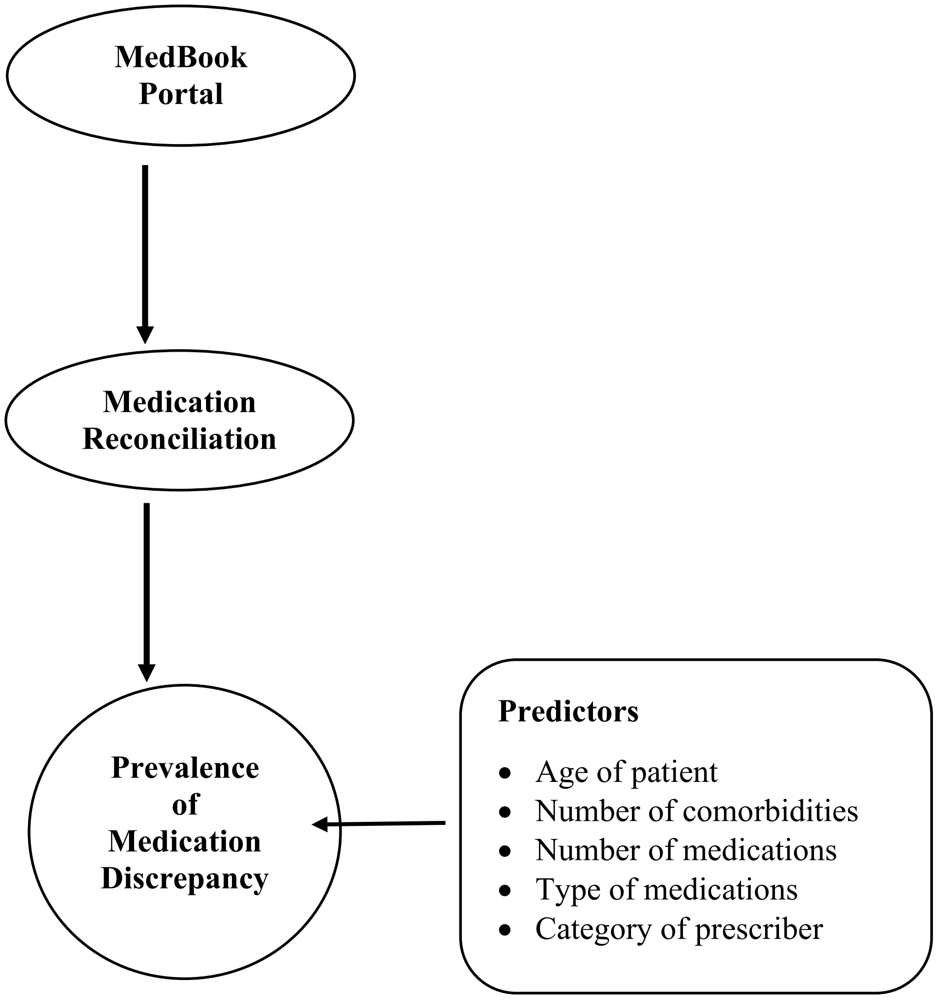

Figure 1.1: Conceptual Framework

1.7 Significance of Research

Proper documentation in hospital discharge summary is essential to ensure continuity of care as patient is involved in multiple care transitions. At hospital discharge, reconciliation is done by checking pre-admission medication list and inpatient medication chart prior to the finalisation of the discharge medication list. Pharmacists serve as the last row of defence to ensure the accuracy of the prescribed medications, couple with discharge counselling to enhance medication understanding and adherence.

In Sarawak, every patient who undergoes medical follow-up at a primary public healthcare setting possesses a personal medical record which will be presented to the doctor during health clinic follow-up and upon hospital admission. Nonetheless, the information upon hospital discharge and clinic follow-up at the secondary or tertiary healthcare setting are not incorporated in the same personal medical record. The discharge summary is provided in separate copies, and the record of clinic follow-up at secondary or tertiary healthcare settings is kept at the clinic. Due to the scattered information, the redundancy of medications with the same or different dosing regimens from various clinics follows up across primary to tertiary healthcare settings is a common issue. Subsequently, this has created confusion and dilemma for the patients as to which regimen to follow, deprived their rights to a holistic treatment approach and jeopardized their health condition.

Recognising the missing part in the healthcare system, enhanced focus on medication reconciliation upon and after hospital discharge could bridge the gap to promote medication and patient safety. By implementing the medication reconciliation upon hospital discharge by pharmacists based in the wards, coupling with patient education and discharge medication plan communication, and followed with the introduction of a medication portal to the prescriber upon discharge which eventually serve as a compilation of personal medication records.

Subsequently, during clinic follow-up, medication reconciliation and updating of the patient medication list in the medication portal will be carried out by the pharmacist in the primary healthcare setting.

In a nutshell, this study may greatly benefit the patients through anticipated medication discrepancies reduction via an innovative intervention. Besides, this intervention is also deemed easy to implement, thus enhancing the duplications and generalizability of the intervention to the healthcare facilities in Malaysia.

1.8 Chapter Summary

Medication reconciliation is one of the key strategies to improve medication safety across transitions of care. Local healthcare providers find it difficult to retrieve previous medical records as the healthcare information system has not been integrated across the healthcare facilities in Malaysia. Thus, an electronic health record that aims to reduce medication discrepancies upon transition of care will be introduced. The study’s findings are essential to healthcare professionals in implementing electronic health records. Due to the limited resources at healthcare facilities, healthcare professionals shall prioritise the patients that need medication reconciliation.

CHAPTER 2

METHODOLOGY

2.1 Study Design

Given the nature of the research question, a randomised controlled trial is developed to compare the prevalence of medication discrepancies in the first prescription upon transition of care from hospitals to primary health clinics between MedBook Portal and standard care. This study is a prospective and multicentre randomised controlled trial with a 1:1 allocation ratio to two groups, namely the intervention group and the control group. MedBook portal is a webpage that enables sharing of patient medication profile among healthcare facilities under the Ministry of Health.

2.2 Operational Definition of Terms

2.2.1 Medication Discrepancies

Medication discrepancy is defined as any variation or difference in medication regimen identified when comparing an old medication list and the newly prescribed medications.

2.2.2 Intentional Medication Discrepancy

Intentional medication discrepancy is defined as a justified discrepancy by a change in the patient's clinical condition.

2.2.3 Unintentional Medication Discrepancy

Unintentional medication discrepancy is defined as unjustified discrepancy after checking with prescriber. It is a medication error, specifically prescribing error.

2.2.4 Types of Medication Discrepancies

Medication discrepancy is classified into:

i. Discrepant dose
ii. Discrepant frequency
iii. Polypharmacy
iv. Drug Omission
v. Drug Addition
vi. Inappropriate drug

Table 2.1 lists the description and example of each type of medication discrepancy identified during medication reconciliation at hospital discharge. Table 2.2 lists the description and example of each type of medication discrepancy identified during medication reconciliation at the first follow-up at health clinics after discharge from the hospital. Figure 2.1 illustrates the example of medication discrepancies detected during medication reconciliation at hospital discharge and the first follow up at the health clinic.

Table 2.1: Description And Example Of Each Type Of Medication Discrepancy Identified During Medication Reconciliation At Hospital Discharge

Type of Medication Discrepancy	Description	Example
Discrepant dose	A different dosage of a medication is prescribed in the discharge prescription compared to the old medication list in the medication history assessment form and inpatient medication charts.	A patient was treated with Atorvastatin 20mg before admission and during admission; however, the patient was discharged with Atorvastatin 40mg.
Discrepant frequency	A different frequency of medication prescribed in the discharge prescription compared to the old medication list in the medication history assessment form and inpatient medication chart.	A patient was treated with Metoprolol 50mg OD before admission. In the ward, the patient has been given Metoprolol 50mg BD and has been discharged with Metoprolol 50mg BD.
Polypharmacy	Multiple agents from the same therapeutic class.	Metoprolol is listed in the medication history assessment form. In the ward, Metoprolol is stopped, and the patient is started with Bisoprolol. During discharge, metoprolol is restarted and given together with Bisoprolol.

Table 2.1 continued

Drug Omission	Medication is not prescribed in the discharge prescription but is listed in the medication history assessment form and inpatient medication chart.	Metoprolol is listed in the medication history assessment form. However, Metoprolol is stopped in the ward and the patient is discharged without Metoprolol.
Drug Addition	Medication is prescribed in the discharge prescription but not listed in the medication history assessment form and inpatient medication chart.	A patient has been discharged with Gliclazide MR. However, Gliclazide MR is not listed in the medication history assessment form or inpatient medication chart.
Inappropriate Drug	Any medication discrepancies that do not fit into any of the previously mentioned categories, such as a medication that no longer needed but is reordered in new prescription; a different formulation of the same item is prescribed.	Example 1:<br><br>A patient had diarrhoea in the ward and was given oral Loperamide 2mg TDS or PRN. However, Loperamide 2mg TDS was written on the discharge prescription when the patient no longer had diarrhoea.<br><br>Example 2:<br><br>A patient has been discharged with Chloramphenicol ear drops. However, the patient has an eye infection and was treated with Chloramphenicol eye drops in the ward.

Table 2.2: Description And Example Of Each Type Of Medication Discrepancy Identified During Medication Reconciliation At The First Follow-Up At Health Clinics After Discharge From The Hospital

Type of Medication Discrepancy	Description	Example
Discrepant dose	A different dosage of a medication is prescribed in the new prescription compared to the discharge prescription.	A patient has been discharged with Losartan 100mg. However, the new prescription on the first visit to the health clinic is Losartan 50mg.
Discrepant frequency	A different frequency of medication prescribed in the new prescription compared to the discharge prescription.	A patient has been discharged with Frusemide 40mg BD. However, only Frusemide 40mg OD is prescribed in the new prescription on the first visit to the health clinic.
Polypharmacy	Multiple agents from the same therapeutic class.	Mefenamic acid and Diclofenac are prescribed concurrently.
Drug Omission	Medication is not prescribed in the new prescription but is listed in the discharge prescription.	A patient was discharged with Metoprolol, Losartan, and Metformin. However, only Metoprolol and Metformin are prescribed in the new prescription on the first visit to the health clinic.

Table 2.2 continued

Drug Addition	Medication is prescribed in the new prescription but not listed in the discharge prescription.	On the first visit to the health clinic, the patient is prescribed with Metoprolol, Losartan and Metformin. However, only Metoprolol and Metformin are listed in the discharge medication list.
Inappropriate Drug	Any medication discrepancies that do not fit into any of the previously mentioned categories, such as a medication that no longer needed but is reordered in new prescription; a different formulation of the same item is prescribed.	Example 1:<br><br>A patient has been discharged with Budesonide inhaler. However, the patient was prescribed with Budesonide nasal spray.<br><br>Example 2:<br><br>A patient has been discharged with Gliclazide 120mg MR OD. However, the patient was prescribed with Gliclazide 120mg OD in the new prescription on the first visit to health clinic.

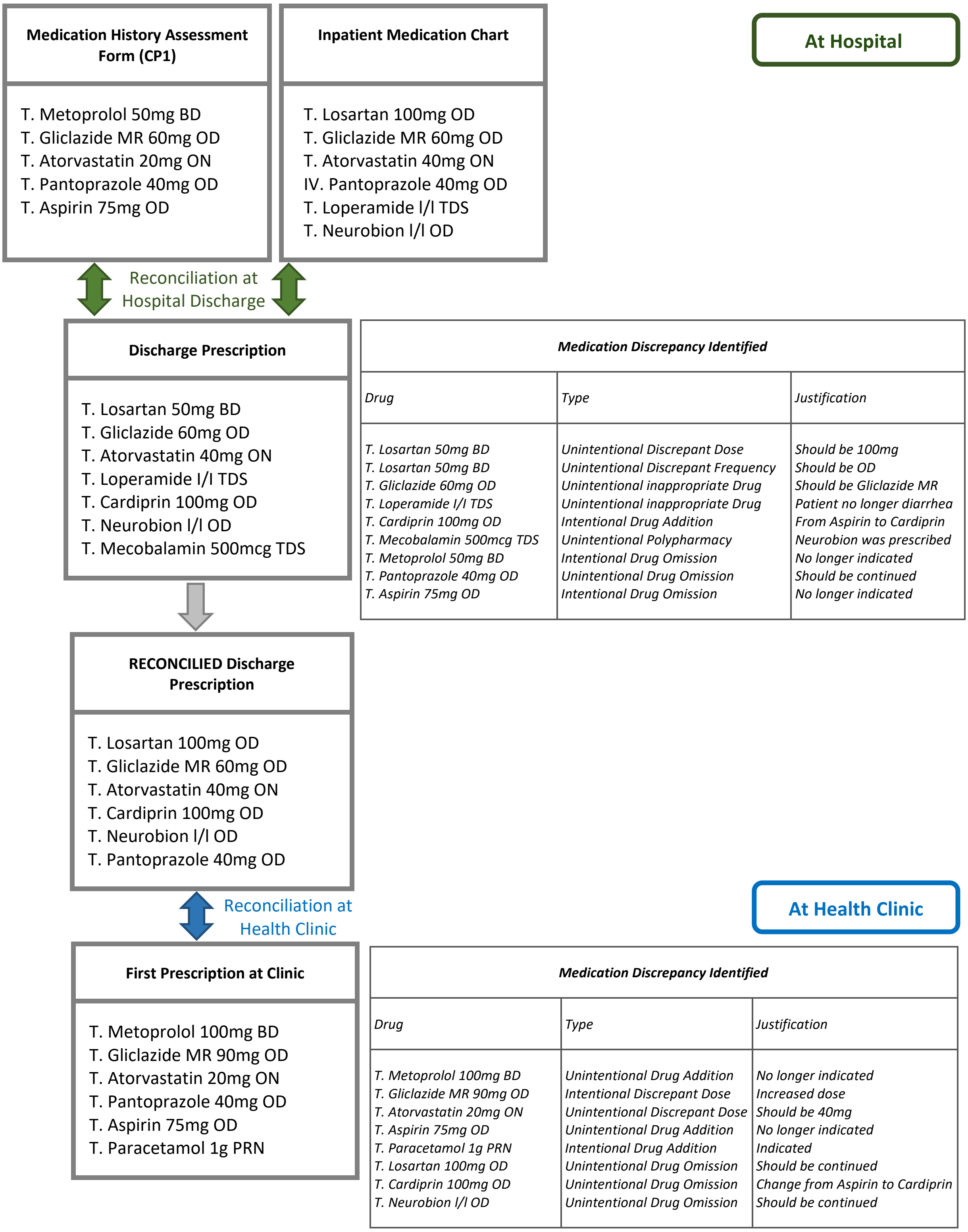

Medication Discrepancy Identified		
Drug	*Type*	*Justification*
T. Losartan 50mg BD	*Unintentional Discrepant Dose*	*Should be 100mg*
T. Losartan 50mg BD	*Unintentional Discrepant Frequency*	*Should be OD*
T. Gliclazide 60mg OD	*Unintentional inappropriate Drug*	*Should be Gliclazide MR*
T. Loperamide I/I TDS	*Unintentional inappropriate Drug*	*Patient no longer diarrhea*
T. Cardiprin 100mg OD	*Intentional Drug Addition*	*From Aspirin to Cardiprin*
T. Mecobalamin 500mcg TDS	*Unintentional Polypharmacy*	*Neurobion was prescribed*
T. Metoprolol 50mg BD	*Intentional Drug Omission*	*No longer indicated*
T. Pantoprazole 40mg OD	*Unintentional Drug Omission*	*Should be continued*
T. Aspirin 75mg OD	*Intentional Drug Omission*	*No longer indicated*

Medication Discrepancy Identified		
Drug	*Type*	*Justification*
T. Metoprolol 100mg BD	*Unintentional Drug Addition*	*No longer indicated*
T. Gliclazide MR 90mg OD	*Intentional Discrepant Dose*	*Increased dose*
T. Atorvastatin 20mg ON	*Unintentional Discrepant Dose*	*Should be 40mg*
T. Aspirin 75mg OD	*Unintentional Drug Addition*	*No longer indicated*
T. Paracetamol 1g PRN	*Intentional Drug Addition*	*Indicated*
T. Losartan 100mg OD	*Unintentional Drug Omission*	*Should be continued*
T. Cardiprin 100mg OD	*Unintentional Drug Omission*	*Change from Aspirin to Cardiprin*
T. Neurobion l/l OD	*Unintentional Drug Omission*	*Should be continued*

Figure 2.1: Example of medication discrepancies detected during medication reconciliation

2.3 Study Population

The targeted population is the adult patients in Sarawak who are discharged from the public tertiary hospitals and referred to the public primary health clinics. These patients will continue to be treated at the selected public primary health clinics.

2.4 Study Sample and Locations

This study will be conducted in four public tertiary hospitals and ten public primary health clinics. The four public hospitals that cater for the patients from southern, central, and northern zones in Sarawak are selected. Those are Sarawak General Hospital (southern zone), Sibu Hospital, Sarikei Hospital (middle zone) and Miri Hospital (northern zone).

During this study, ten public primary health clinics which are located nearby to the four selected hospitals are chosen to follow up the participants who will be discharged and referred to these clinics. The healthcare facilities involved in this study are presented in Table 2.3. All patients who fulfil the selection criteria mentioned later will be invited to participate in the study.

Table 2.3: Public Major Hospitals and Health Clinics Involved in this Study

Public Major Hospitals	Public Primary Health Clinics
Sarawak General Hospital	Batu Kawa HC, Kota Sentosa HC, Kota Samarahan HC
Sibu Hospital	Jalan Oya HC, Lanang HC, Sibu Jaya HC
Miri Hospital	Miri HC, Tudan HC
Sarikei Hospital	Sarikei HC, Bintangor HC

2.4.1 Inclusion criteria

Patients who fulfil the following criteria will be invited to participate in the study:

i. Adult patients aged 18 years and above in general medical wards who had any of the following comorbidities: (1) cardiovascular related diseases, e.g.: hypertension, acute coronary syndrome, heart failure, stroke, atrial fibrillation; (2) endocrine related diseases, e.g.: diabetes, thyroid disorders; (3) renal related diseases, e.g.: chronic kidney disease, end stage renal failure; (4) respiratory related diseases, e.g.: asthma, chronic obstructive pulmonary disease.

ii. Patients who are referred to the selected primary health clinics after hospital discharge.

iii. Patients with home-based medical card.

2.4.2 Exclusion criteria

Patients discharged during public holidays or weekends will be excluded from this study.

2.4.3 Withdrawal criteria

Patients readmitted to hospitals before the first review at the health clinic shall be withdrawn from the study. Patients can choose to withdraw at any time. Withdrawn patients will not be replaced.

2.5 Sample Size Calculation

This study aims to compare the prevalence of medication discrepancies in the first prescription upon transition of care from hospitals to primary health clinics between MedBook Portal and standard care. Hence, a two-tailed z-test of proportion between two groups with 80% power and a 5% of level significance will be used (10). The prevalence of medication discrepancies upon discharge reported in a previous study is 49% (8). We estimate the reduction of medication discrepancies via implementation of MedBook is 15%. Thus, a sample size of 332 subjects, 166 subjects in each group, is sufficient to detect a clinically importance difference of 15% between groups. However, with the estimation of a 20% loss to follow up, a total of 398 subjects will be recruited.

In addition, this study also aims to ascertain the prevalence of medication discrepancies and type of medication discrepancies in discharge prescription and in the first prescription upon transition of care from hospitals to primary health clinics. Sample size is calculated by using the following formula (11):

$$n = \frac{Z^2 P(1-P)}{d^2}$$

where n = sample size,

Z = Z statistic for a level of confidence,

P = expected prevalence or proportion

d = precision

By determining the Z = 1.96 for 95% CI and d = 0.05, the minimum sample size is 384. Based on the above sample size calculation, a total of 398 patients will be recruited. The sample size allocation for each hospital is determined on a pro-rata basis based on the total bed numbers in general medical wards and Bed Occupancy Rate (Table 2.4).

Table 2.4: Sample Size Allocation Of Each Hospitals

Public Major Hospitals	Wards	Total Bed Numbers	Bed Occupancy Rate (%)	Sample Size Allocation
Sarawak General Hospital	Male General Ward	48	114.4	148
	Female General Ward	48	102.0	
Sibu Hospital	Male General Ward 1	36	62.2	110
	Male General Ward 2	36	73.4	
	Female General Ward	32	83.5	
Miri Hospital	Male General Ward	29	114.1	74
	Female General Ward	30	62.8	
Sarikei Hospital	Male General Ward	35	82.4	66
	Female General Ward	35	50.2	
Total	-	329	-	398

2.6 Study Duration

This study is estimated to be conducted over 6 months, expecting to commence in August 2023.

2.7 Data and Instruments

2.7.1 Data Collection Form

Medication discrepancies detected will be categorised according to the following groupings, discrepant dose, discrepant frequency, polypharmacy, drug omission, drug addition and inappropriate drug. Other data that will be collected include age, gender, number of medications, type of medication based on Anatomical Therapeutic Chemical (ATC) categorisation in Drug Formulary Ministry of Health Malaysia, comorbidities, and category of prescriber.

There are two electronic data collection forms using Google Form for data entry. The first data collection form (Appendix A) is administered by pharmacist at hospital before allocation. The second data collection form (Appendix B) is to be administered by pharmacist at the health clinic.

2.7.2 MedBook Portal

MedBook portal (https://aplikasi.jknsarawak.moh.gov.my/medbook) is a webpage that enable sharing of patient medication records among healthcare facilities under Ministry of Health. Sarawak State Health Department owned and managed this portal. All the information is kept under their server. Doctors in the Health Clinics and Study Pharmacists will be given access to this portal whereby each of the healthcare providers shall log in using their Identification Number and the password created by them. Doctor shall log in to MedBook Portal to check on discharge medication list when reviewing the patient at health clinic. MedBook Portal can be accessed both using a smartphone or a computer. The design of MedBook Portal in the smartphone version is shown in Figure 2.2. The design of MedBook Portal in the computer version is shown in Figure 2.3.

During the discharge process, the study pharmacist at hospital will upload the discharge prescription to MedBook Portal for both the control group and intervention group. When uploading the discharge prescription, the study pharmacist shall select the radio button ‘intervention’ for patients in the intervention group. MedBook Portal allows doctors at health clinics to view the prescription under the intervention group only. Whereas pharmacists at health clinics can view all the prescriptions in both groups. This setting is done based on the user role in MedBook Portal. The purpose of the setting is to ease the study pharmacist at the health clinic to compare the discharge prescription in MedBook Portal and the first prescription at the health clinic to identify medication discrepancies in both groups.

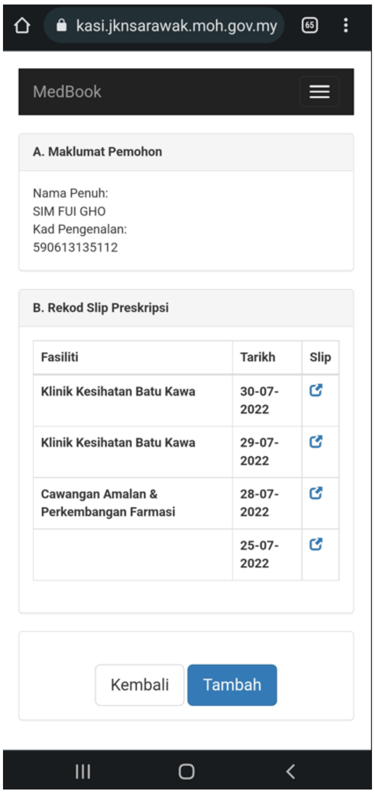

Figure 2.2: MedBook Portal in smartphone version

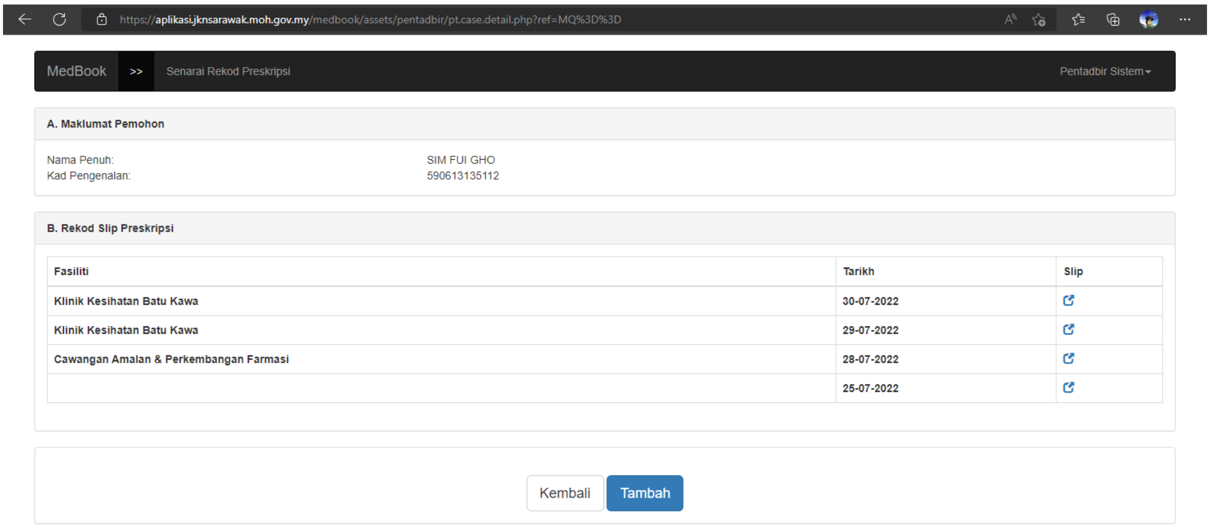

Figure 2.3: MedBook Portal in computer version

2.7.3 MedBook Portal Notice

A MedBook Portal Notice will be placed in front of the patient's home-based medical card. Only patients in the intervention group are given MedBook Portal Notice. Hence, the doctors and pharmacists at health clinics can identify the recruited patients in the intervention group. The layout of the MedBook Portal Notice is shown in Figure 2.4.

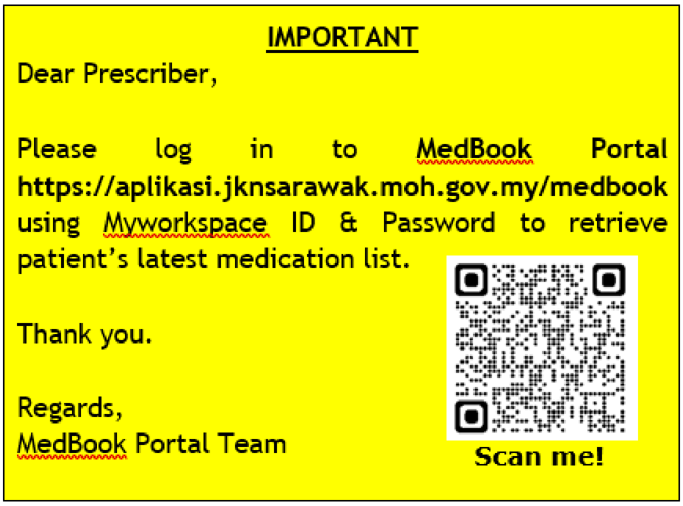

Figure 2.4: MedBook Portal Notice

2.8 Recruitment

In this study, patients who are decided to be discharged from the selected hospital and fulfilled the inclusion criteria will be recruited. The patients will be given a full explanation regarding the study according to the patient information sheet before enrolment. Subsequently, patients will be requested to provide written informed consent (Appendix C) if they agree to participate in the study.

2.9 Medication Reconciliation Upon Hospital Discharge

Medications in the discharge prescription will be considered as the primary source of reference for discharge reconciliation, as this is the list of medications that the pharmacy will refer to for medication preparation and eventually will be given to the patients upon discharge. Study pharmacists will conduct discharge medication reconciliation before randomization. Discharge medication reconciliation will be carried out by comparing the medications in the discharge prescription against the medications in the medication history assessment form (pre-admission medications) and inpatient medication charts (medications started during admission) to detect medication discrepancies before discharge medications preparation. If any differences are detected, study pharmacists will confirm with the doctor to decide intentional or unintentional medication discrepancies-any amendment to be documented on the discharge prescription.

2.10 Randomisation and blinding

The recruited patients will be randomised to the intervention and control groups in a 1: 1 ratio before discharge. Randomisation will be conducted using the online program available at http://www.graphpad.com/quickcalcs/index.cfm (12). A research coordinator will be appointed to administer the randomisation and inform the study pharmacist to deliver the intervention according to the assigned group. The research coordinator has no contact with patients, study pharmacists and doctors. In addition, the research coordinator will manage the

patient list with their code and group assignment. Subsequently, the research coordinator will send the patient list to the principal investigator to remind the patient of the follow-up date at the health clinic. Due to the nature of the intervention, patients, doctors, and study pharmacists are not blinded.

2.11 Intervention Group

MedBook Portal Notice will be attached in front of the patient's home-based medical card for the doctors and pharmacists at health clinics to identify the recruited patients. This appointment would be their first follow-up at health clinics after discharge from the hospital.

During the doctor's review of the patient in the intervention group, the doctor shall log in to the MedBook Portal to check on the discharge medication list and review the case based on the previous medical record in the home-based medical card and the discharge note if the patient hand over it to the doctors in their first review. Then, the doctor will conduct medication reconciliation by comparing the list of medications in the discharge prescription against the new prescription prepared.

Study pharmacists at the health clinics are required to screen patients' home-based medical cards presented to the pharmacy upon collecting medications. Once the pharmacists at health clinics identify the recruited patients via the MedBook Portal Notice, the pharmacist shall log in to the MedBook Portal to check the discharge medication list. The pharmacist shall conduct medication reconciliation by comparing the new prescription received against the discharge medication list in the portal. Intervention is to be carried out by the study pharmacists through confirmation with the doctor to determine the intentional or unintentional medication discrepancies.

2.12 Control Group

Patients will receive the standard care in which doctors review patients based on the previous medical record in the home-based medical card and discharge note if the patient hands it to the doctors in their first review. Doctors cannot retrieve a patient's discharge medication list from MedBook Portal. No MedBook Portal Notice is attached to the patient's home-based medical card.

The principal investigator will provide a patient list in the control group to the study pharmacist at the health clinic. By knowing their appointment date, the study pharmacist shall search for the first prescription upon transitioning care from the hospital to the primary health clinic. Subsequently, the study pharmacist shall compare the discharge prescription in MedBook Portal with the first prescription to identify medication discrepancies. If any differences are detected, study pharmacists will confirm with the doctor to decide intentional or unintentional medication discrepancies. However, the study pharmacist might not be able to retrieve the first prescription immediately on the appointment date as there is no MedBook Portal Notice attached. As a result, the study pharmacist can only check with the prescriber on the discrepancy at a later time.

2.13 Data Collection

The prevalence of medication discrepancies during discharge is collected through medication reconciliation by the study pharmacist at the hospital using the electronic data collection form. In addition, the type of medication discrepancies and predictors associated with medication discrepancies will be collected simultaneously using the first data collection form (Appendix A) by the study pharmacist at a hospital before allocation.

To compare the prevalence of medication discrepancies in the first prescription upon transition of care from hospitals to primary health clinics between the MedBook Portal and standard care,

data collection on the number of medication discrepancies will be carried out during the first follow-up at the health clinic after the hospital discharge. A pharmacist will administer the second data collection form (Appendix B) at the health clinic.

Throughout the study, there is only one follow-up visit at the health clinic, the first after hospital discharge. However, the time frame between the discharge date and the appointment date for the first follow-up at the health clinic varies from days to weeks depending on the duration determined by the hospital. Thus, the expected duration of subject participation would range from one week to two months.

To reduce loss to follow-up, the study pharmacist shall contact the patient via WhatsApp or phone call to remind the patient of the follow-up date at the health clinic. The schedule of enrolment, interventions and assessments of the study are presented in Table 2.5. The study flow chart is illustrated in Figure 2.5.

Table 2.5: Schedule of Enrolment, Interventions and Assessments

**Timepoint**	**Study Period**			
	**Enrolment**	**Allocation**	**Post-allocation**	**Close-out**
	**-t_1**	**0**	**1st Primary Health Clinic Follow Up**	
ENROLMENT:				
Eligibility screen	X			
Participant Information Sheet	X			
Informed consent	X			
Allocation		X		
INTERVENTIONS:				
MedBook Portal		X	X	
ASSESSMENTS:				
Prevalence of medication discrepancies	X		X	
Predictors of medication discrepancies	X		X	

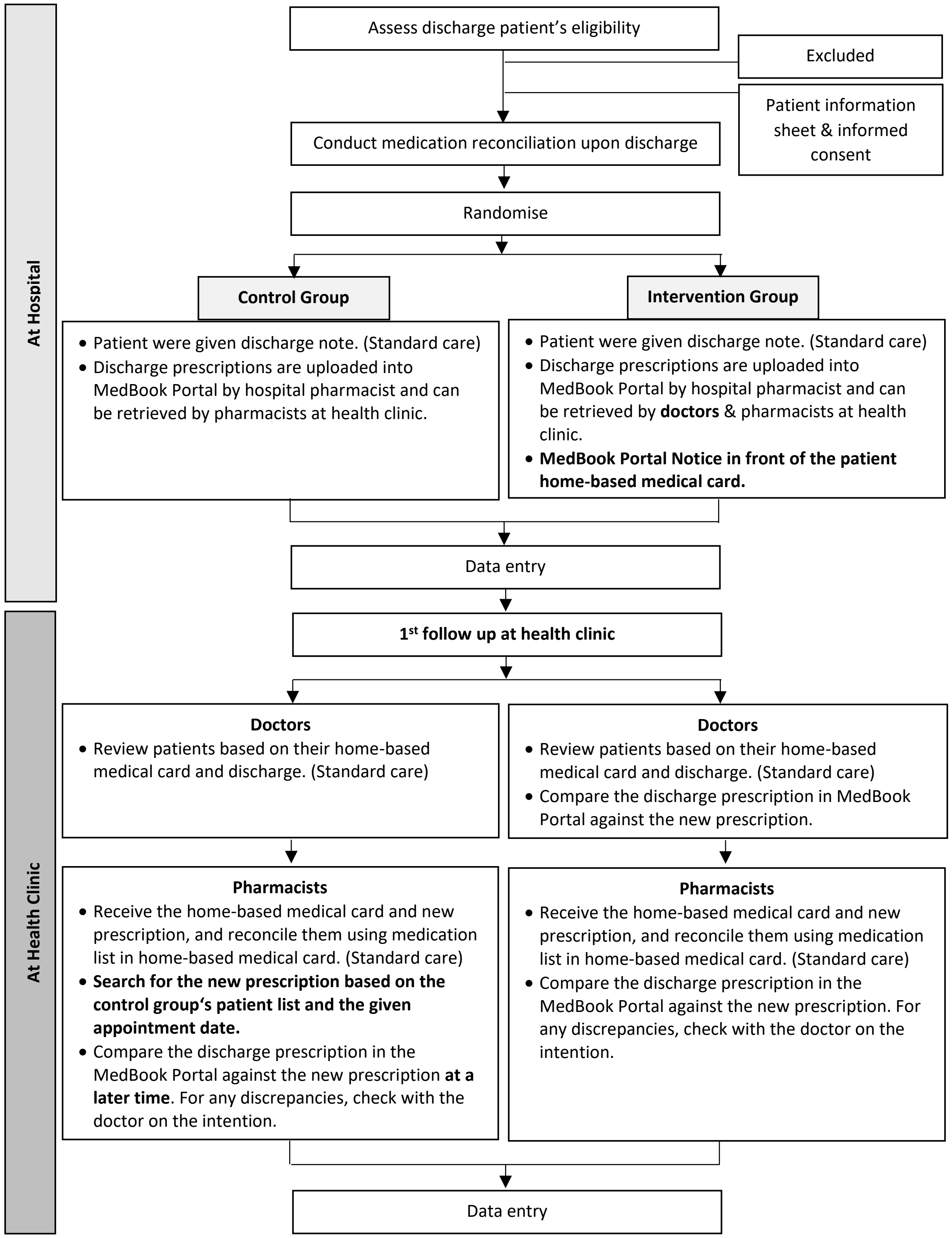

Figure 2.5: Study Flow Chart

2.14 Outcomes

The primary outcome of the study is the percentage of prescriptions with medication discrepancies in the first prescription during the first follow-up visit at the primary health clinic after hospital discharge. A comparison of the prevalence of prescriptions with medication discrepancies in the intervention group and the control group will be conducted.

The secondary outcome of the study is the percentage of prescriptions with medication discrepancies in the discharge prescription. This is measured before the intervention. In addition, the type of medication discrepancies and predictors associated with medication discrepancies in discharge prescriptions and the first prescriptions upon transitioning care from hospitals to primary health clinics will be collected. Predictors associated with medication discrepancies will be analysed using multiple logistic regression.

2.15 Data Analysis

All data will be analysed using The jamovi project (2022) (Version 2.3). Descriptive statistics (frequency and percentage; mean and standard deviation) will be used to describe the socio-demographic characteristics of the participants.

Categorical variables, expressed as numbers and percentages, are compared by the Chi-square test. Continuous variables will be tested for normality using One-Sample Kolmogorov-Smirnov Test and compared using independent t-test for normally distributed data and the Mann-Whitney U test for non-normally distributed data. Data with normal distribution are reported as mean (standard deviation, SD), while non-normally distributed variables are reported as median (interquartile range, IQR).

The predictors associated with medication discrepancies will first be analysed using univariable logistic regression. The variables with p-value <0.1 by univariable analysis will be included in the multivariable logistic regression model. All statistical tests are two-tailed and $p<0.05$ is set to be statistically significant.

2.16 Chapter Summary

This is a two-group randomised controlled trial conducted at four public tertiary hospitals and ten public primary health clinics in Sarawak, Malaysia. Adult patients in general medical wards who are discharged from the hospitals and referred to the selected primary health clinics will be recruited in the study. Eligible subjects will be randomised into intervention group and control group in a ratio of 1:1. During the first follow-up at the primary health clinic after discharge from hospital, doctors in the primary health clinics review the patients in the intervention group based on the discharge medication list in the MedBook Portal. Patients in the control group receive the standard care. Study pharmacists conduct medication reconciliation and identify medication discrepancies.

The primary outcome of this study is the percentage of prescriptions with medication discrepancies in the first prescription during the first follow-up visit at the primary health clinic after hospital discharge. A comparison of the prevalence of prescriptions with medication discrepancies in the intervention group and the control group will be conducted. The secondary outcomes are the percentage of prescriptions with medication discrepancies in the discharge prescription, the type of medication discrepancies and predictors associated with medication discrepancies in discharge prescriptions and the first prescriptions upon transitioning care from hospitals to primary health clinics. All data will be analysed using The jamovi project (2022) (Version 2.3). All statistical tests are two-tailed and $p<0.05$ is set to be statistically significant.

CHAPTER 3

GANTT CHART

3.1 Gantt Chart

A Gantt Chart illustrates the duration of the task in the study and the sequencing of each activity. Proper planning for each activity is crucial to ensure the study is completed in the allocated time. The main activity of this study is depicted in Table 3.1.

Table 3.1: Gantt Chart

Activities	2022						2023												2024										
	7	8	9	10	11	12	1	2	3	4	5	6	7	8	9	10	11	12	1	2	3	4	5	6	7	8	9	10	11
**Literature Review**	■	■	■	■	■	■	■	■	■	■	■	■	■	■	■	■	■	■	■	■	■	■	■	■	■	■	■	■	
**Proposal Write-Up**	■	■	■	■	■	■	■	■																					
**Study Site Meeting**		■	■	■	■	■	■	■	■	■																			
**Ethics submission & approval**										■	■	■	■																
**Preparation of study materials & training**													■	■															
**Data Collection**														■	■	■	■	■	■	■									
**Data Analysis**																				■	■	■							
**Write Up**																						■	■	■	■	■	■	■	
**Submission for publication**																											■		

REFERENCES

1. Medication Safety in Transitions of Care. Geneva: World Health Organization; 2019. Report No.: WHO/UHC/SDS/2019.9.

2. Abu Farha R, Yousef A, Gharaibeh L, Alkhalaileh W, Mukattash T, Alefishat E. Medication discrepancies among hospitalized patients with hypertension: assessment of prevalence and risk factors. BMC Health Serv Res. 2021;21(1):1338.

3. Cornu P, Steurbaut S, Leysen T, De Baere E, Ligneel C, Mets T, et al. Effect of medication reconciliation at hospital admission on medication discrepancies during hospitalization and at discharge for geriatric patients. Ann Pharmacother. 2012;46(4):484-94.

4. Hias J, Van der Linden L, Spriet I, Vanbrabant P, Willems L, Tournoy J, et al. Predictors for unintentional medication reconciliation discrepancies in preadmission medication: a systematic review. Eur J Clin Pharmacol. 2017;73(11):1355-77.

5. Farley TM, Shelsky C, Powell S, Farris KB, Carter BL. Effect of clinical pharmacist intervention on medication discrepancies following hospital discharge. Int J Clin Pharm. 2014;36(2):430-7.

6. Kripalani S, Roumie CL, Dalal AK, Cawthon C, Businger A, Eden SK, et al. Effect of a pharmacist intervention on clinically important medication errors after hospital discharge: a randomized trial. Ann Intern Med. 2012;157(1):1-10.

7. Hassali MA, Al-Haddad M, Shafie AA, Tangiisuran B, Saleem F, Atif M, et al. Perceptions among general medical practitioners toward implementation of medication reconciliation program for patients discharged from hospitals in Penang, Malaysia. J Patient Saf. 2012;8(2):76-80.

8. Law BK, Chong CP. Prevalence and predictors of medication discrepancies upon discharge among adult patients: a prospective study from Malaysia. Journal of Asian Association of Schools of Pharmacy. 2017;6(1):1-9.

9. Mueller SK, Kripalani S, Stein J, Kaboli P, Wetterneck TB, Salanitro AH, et al. A toolkit to disseminate best practices in inpatient medication reconciliation: multi-center medication reconciliation quality improvement study (MARQUIS). Jt Comm J Qual Patient Saf. 2013;39(8):371-82.

10. Sakpal TV. Sample size estimation in clinical trial. Perspect Clin Res. 2010;1(2):67-9.

11. Naing L, Winn T, Rusli BN. Practical Issues in Calculating the Sample Size for Prevalence Studies Archives of Orofacial Sciences. 2006;1:9-14.

12. Suresh K. An overview of randomization techniques: An unbiased assessment of outcome in clinical research. J Hum Reprod Sci. 2011;4(1):8-11.

APPENDICES

Appendix A: Data Collection Form to be administered by Pharmacist at Hospital (https://forms.gle/wZrjjTpgLtHM8cb59)

MedRecon Data collection Form (Hospital)

*Required

1. ID *

   This ID is the ID assigned during the recruitment of the patient

2. Gender *

   Mark only one oval.

   - Male
   - Female

3. Age *

4. Discharged from *

   Mark only one oval.

   - Hospital Umum Sarawak
   - Hospital Sibu
   - Hospital Miri
   - Hospital Sarikei

5. Refer to *

   Mark only one oval.

   - KK Sentosa
   - KK Batu Kawa
   - KK Samarahan
   - KK Jalan Oya
   - KK Lanang
   - KK Sibu Jaya
   - KK Miri
   - KK Tudan
   - KK Sarikei
   - KK Bintangor

6. Comorbidities as per case note (Please list down refer) *

7. Number of Medication *

8. Category of prescriber *

   Mark only one oval.

   - House Officer
   - Medical Officer
   - Consultant/ Specialist

9. Any other Unintentional Medication Discrepancies? *

Mark only one oval.

- Yes
- No

Drug #1

10. Drug Name with Unintentional Medication Discrepancy *

____

11. Type of Medication Discrepancies *

Mark only one oval.

- Discrepant dose
- Discrepant frequency
- Polypharmacy
- Drug Omission
- Drug Addition
- Inappropriate Drug

12. Any other Unintentional Medication Discrepancies? *

Mark only one oval.

- Yes
- No

Drug #2

13. Drug Name with Unintentional Medication Discrepancy *

____

14. Type of Medication Discrepancies *

Mark only one oval.

- Discrepant dose
- Discrepant frequency
- Polypharmacy
- Drug Omission
- Drug Addition
- Inappropriate Drug

15. Any other Unintentional Medication Discrepancies? *

Mark only one oval.

- Yes
- No

Drug #3

16. Drug Name with Unintentional Medication Discrepancy *

____

17. Type of Medication Discrepancies *

Mark only one oval.

- Discrepant dose
- Discrepant frequency
- Polypharmacy
- Drug Omission
- Drug Addition
- Inappropriate Drug

18. Any other Unintentional Medication Discrepancies? *

Mark only one oval.

- Yes
- No

Drug #4

19. Drug Name with Unintentional Medication Discrepancy *

20. Type of Medication Discrepancies *

Mark only one oval.

- Discrepant dose
- Discrepant frequency
- Polypharmacy
- Drug Omission
- Drug Addition
- Inappropriate Drug

21. Any other Unintentional Medication Discrepancies? *

Mark only one oval.

- Yes
- No

Drug #5

22. Drug Name with Unintentional Medication Discrepancy *

23. Type of Medication Discrepancies *

Mark only one oval.

- Discrepant dose
- Discrepant frequency
- Polypharmacy
- Drug Omission
- Drug Addition
- Inappropriate Drug

24. Any other Unintentional Medication Discrepancies? *

Mark only one oval.

- Yes
- No

Drug #6

25. Drug Name with Unintentional Medication Discrepancy *

26. Type of Medication Discrepancies *

Mark only one oval.

- Discrepant dose
- Discrepant frequency
- Polypharmacy
- Drug Omission
- Drug Addition
- Inappropriate Drug

27. Any other Unintentional Medication Discrepancies? *

Mark only one oval.

- ◯ Yes
- ◯ No

Drug #7

28. Drug Name with Unintentional Medication Discrepancy *

____

29. Type of Medication Discrepancies *

Mark only one oval.

- ◯ Discrepant dose
- ◯ Discrepant frequency
- ◯ Polypharmacy
- ◯ Drug Omission
- ◯ Drug Addition
- ◯ Inappropriate Drug

30. Any other Unintentional Medication Discrepancies? *

Mark only one oval.

- ◯ Yes
- ◯ No

Drug #8

31. Drug Name with Unintentional Medication Discrepancy *

____

32. Type of Medication Discrepancies *

Mark only one oval.

- ◯ Discrepant dose
- ◯ Discrepant frequency
- ◯ Polypharmacy
- ◯ Drug Omission
- ◯ Drug Addition
- ◯ Inappropriate Drug

33. Any other Unintentional Medication Discrepancies? *

Mark only one oval.

- ◯ Yes
- ◯ No

Drug #9

34. Drug Name with Unintentional Medication Discrepancy *

____

35. Type of Medication Discrepancies *

Mark only one oval.

- ◯ Discrepant dose
- ◯ Discrepant frequency
- ◯ Polypharmacy
- ◯ Drug Omission
- ◯ Drug Addition
- ◯ Inappropriate Drug

36. Any other Unintentional Medication Discrepancies? *

Mark only one oval.

- ( ) Yes
- ( ) No

Drug #10

37. Drug Name with Unintentional Medication Discrepancy *

____

38. Type of Medication Discrepancies *

Mark only one oval.

- ( ) Discrepant dose
- ( ) Discrepant frequency
- ( ) Polypharmacy
- ( ) Drug Omission
- ( ) Drug Addition
- ( ) Inappropriate Drug

This content is neither created nor endorsed by Google.

Google Forms

Appendix B: Data Collection Form to be administered by Pharmacist at Health Clinic (https://forms.gle/3wENridxTmN9WYV77)

MedRecon Data collection Form (Health Clinic)

*Required

1. ID *

This ID is the ID assigned during the recruitment of the patient

2. Referred to *

Mark only one oval.

- KK Sentosa
- KK Batu Kawa
- KK Samarahan
- KK Jalan Oya
- KK Lanang
- KK Sibu Jaya
- KK Miri
- KK Tudan
- KK Sarikei
- KK Bintangor

3. Number of Medication *

Mark only one oval.

- Medical Officers
- Family Physician Specialist
- Consultant/ Specialist
- Medical Assistant
- Nurse

5. Any Medication Discrepancies? *

Mark only one oval.

- Yes
- No

Drug #1

6. Drug Name with Discrepancies *

7. Intentional or unintentional *

Mark only one oval.

- Intentional
- Unintentional

8. Type of Medication Discrepancies *

Mark only one oval.

- ◯ Discrepant Dose
- ◯ Discrepant Frequency
- ◯ Polypharmacy
- ◯ Drug Omission
- ◯ Drug Addition
- ◯ Inappropriate Drug

9. Any other Medication Discrepancies? *

Mark only one oval.

- ◯ Yes
- ◯ No

Drug #2

10. Drug Name with Discrepancies *

____

11. Intentional or unintentional *

Mark only one oval.

- ◯ Intentional
- ◯ Unintentional

12. Type of Medication Discrepancies *

Mark only one oval.

- ◯ Discrepant Dose
- ◯ Discrepant Frequency
- ◯ Polypharmacy
- ◯ Drug Omission
- ◯ Drug Addition
- ◯ Inappropriate Drug

13. Any other Medication Discrepancies? *

Mark only one oval.

- ◯ Yes
- ◯ No

Drug #3

14. Drug Name with Discrepancies *

____

15. Intentional or unintentional *

Mark only one oval.

- ◯ Intentional
- ◯ Unintentional

16. Type of Medication Discrepancies *

Mark only one oval.

- Discrepant Dose
- Discrepant Frequency
- Polypharmacy
- Drug Omission
- Drug Addition
- Inappropriate Drug

17. Any other Medication Discrepancies? *

Mark only one oval.

- Yes
- No

Drug #4

18. Drug Name with Discrepancies *

____

19. Intentional or unintentional *

Mark only one oval.

- Intentional
- Unintentional

20. Type of Medication Discrepancies *

Mark only one oval.

- Discrepant Dose
- Discrepant Frequency
- Polypharmacy
- Drug Omission
- Drug Addition
- Inappropriate Drug

21. Any other Medication Discrepancies? *

Mark only one oval.

- Yes
- No

Drug #5

22. Drug Name with Discrepancies *

____

23. Intentional or unintentional *

Mark only one oval.

- Intentional
- Unintentional

24. Type of Medication Discrepancies *

Mark only one oval.

- ◯ Discrepant Dose
- ◯ Discrepant Frequency
- ◯ Polypharmacy
- ◯ Drug Omission
- ◯ Drug Addition
- ◯ Inappropriate Drug

25. Any other Medication Discrepancies? *

Mark only one oval.

- ◯ Yes
- ◯ No

Drug #6

26. Drug Name with Discrepancies *

____

27. Intentional or unintentional *

Mark only one oval.

- ◯ Intentional
- ◯ Unintentional

28. Type of Medication Discrepancies *

Mark only one oval.

- ◯ Discrepant Dose
- ◯ Discrepant Frequency
- ◯ Polypharmacy
- ◯ Drug Omission
- ◯ Drug Addition
- ◯ Inappropriate Drug

29. Any other Medication Discrepancies? *

Mark only one oval.

- ◯ Yes
- ◯ No

Drug #7

30. Drug Name with Discrepancies *

____

31. Intentional or unintentional *

Mark only one oval.

- ◯ Intentional
- ◯ Unintentional

32. Type of Medication Discrepancies *

Mark only one oval.

- Discrepant Dose
- Discrepant Frequency
- Polypharmacy
- Drug Omission
- Drug Addition
- Inappropriate Drug

33. Any other Medication Discrepancies? *

Mark only one oval.

- Yes
- No

Drug #8

34. Drug Name with Discrepancies

____

35. Intentional or unintentional *

Mark only one oval.

- Intentional
- Unintentional

36. Type of Medication Discrepancies *

Mark only one oval.

- Discrepant Dose
- Discrepant Frequency
- Polypharmacy
- Drug Omission
- Drug Addition
- Inappropriate Drug

37. Any other Medication Discrepancies? *

Mark only one oval.

- Yes
- No

Drug #9

38. Drug Name with Discrepancies *

____

39. Intentional or unintentional *

Mark only one oval.

- Intentional
- Unintentional

40. Type of Medication Discrepancies *

Mark only one oval.

- ◯ Discrepant Dose
- ◯ Discrepant Frequency
- ◯ Polypharmacy
- ◯ Drug Omission
- ◯ Drug Addition
- ◯ Inappropriate Drug

41. Any other Medication Discrepancies? *

Mark only one oval.

- ◯ Yes
- ◯ No

Drug #10

42. Drug Name with Discrepancies *

____

43. Intentional or unintentional *

Mark only one oval.

- ◯ Intentional
- ◯ Unintentional

44. Type of Medication Discrepancies *

Mark only one oval.

- ◯ Discrepant Dose
- ◯ Discrepant Frequency
- ◯ Polypharmacy
- ◯ Drug Omission
- ◯ Drug Addition
- ◯ Inappropriate Drug

This content is neither created nor endorsed by Google.

Google Forms

Appendix C: Study Participant Information Sheet and Informed Consent Form in English

PATIENT INFORMATION SHEET AND INFORMED CONSENT FORM

(for adult subjects and interventional studies)

1. **Title of study**:
A Randomized Controlled Trial to Compare MedBook Portal and Standard Care in Medication Reconciliation at Transitions of Care from Hospital Discharge to Primary Healthcare Settings

2. **Name of investigator and institution:**
Phang Yen Yen, Pharmaceutical Services Division, Sarawak State Health Department.
Professor Dr. Kuan Jew Win, Faculty of Medicine and Health Sciences, Universiti Malaysia Sarawak

3. **Name of sponsor**:
Pharmaceutical Services Division, Sarawak State Health Department.

4. **Introduction:**
You are invited to participate in a research study because you will be discharged and will be referred to the health clinics for follow up treatment. The details of the research trial are
described in this document. It is important that you understand why the research is being done and what it will involve. Please take your time to read through and consider this information carefully before you decide if you are willing to participate. Ask the study staff if anything is unclear or if you like more information. After you are properly satisfied that you understand this study, and that you wish to participate, you must sign this informed consent form.

Your participation in this study is voluntary. You do not have to be in this study if you do not want to. You may also refuse to answer any questions you do not want to answer. If you volunteer to be in this study, you may withdraw from it at any time. If you withdraw, any data collected from you up to your withdrawal will still be used for the study. Your refusal to participate or withdrawal will not affect any medical or health benefits to which you are otherwise entitled.

This study has been approved by the Medical Research and Ethics Committee, Ministry of Health Malaysia.

5. **What is the purpose of the study?**
The purpose of this study is to examine the effectiveness of an intervention, named as MedBook Portal, in reducing medication discrepancies upon transition of care from hospitals to health clinics. MedBook portal is a webpage that enable sharing of patient medication profile among healthcare facilities under Ministry of Health. MedBook Portal
aims to promote medication and patient safety.

A total of 398 subjects like you from the four public hospitals will be participating in this study. The four hospitals are Sarawak General Hospital, Sibu Hospital, Sarikei Hospital and Miri Hospital. The subjects will be discharged and referred to the ten public primary health clinics, namely, Batu Kawa Health Clinic, Kota Sentosa Health Clinic, Kota Samarahan Health Clinic, Jalan Oya Health Clinic, Lanang Health Clinic, Sibu Jaya Health Clinic, Miri Health Clinic, Tudan Health Clinic, Sarikei Health Clinic and Bintangor Health Clinic.

The whole study will last about 6 months and your participation will be about 1 week to 2 months depending on your appointment date at the health clinic.

Page **1** of **4**

6. **What kind of study products will I receive?**
If you agree to participate in the study, you will be randomly (by chance, like flipping a coin) assigned to one of the treatment groups below. You have equal chance of being assigned to each of the groups.

Group 1:
Your discharge prescription will be uploaded to MedBook Portal which will be accessed by your healthcare providers at the health clinic. MedBook Portal Notice will be attached on your home-based medical card.
After discharge from hospital, you are required to follow at health clinic according to the appointment date. This would be the first follow-up at the health clinic after discharge from hospital. During this appointment, you shall bring over your home-based medical card with the MedBook Portal Notice attached and any documents that are informed by the hospital or health clinic to your doctor and pharmacist. Your doctor will review your case based on your discharge prescription in MedBook Portal, your previous medical record in the home-based medical card and any documents handed over to he or she. Your doctor and pharmacist will conduct medication reconciliation by comparing the discharge prescription in MedBook Portal against the new prescription prepared at the health clinic.

Group 2:
You will be given usual care, in which there is no MedBook Portal and no MedBook Portal Notice provided.
After discharge from hospital, you are required to follow at health clinic according to the appointment date. This would be the first follow-up at the health clinic after discharge from hospital. During this appointment, you shall bring over your home-based medical card and any documents that are informed by the hospital or health clinic to your doctor and pharmacist. Your doctor will review your case based on your previous medical record in the home-based medical card and any documents handed over to he or she.

In order to examine the effectivenss of MedBook Portal, the investigator will compare the percentage of prescription with medication discrepancies between both group using statistical tools.

7. **What will happen if I decide to take part?**
You will have to attend the first follow-up at health clinic You will be contacted by the investigator via WhatsApp or phone call to remind you on the follow up date at health clinic. Investigator will contact you via the telephone number 082-473200 or 01129060439.

8. **What are my responsibilities when taking part in this study?**
It is important that you answer all of the questions asked by the study staff honestly and completely. If your condition or circumstances change during the study, you must tell the investigator. You shall bring over your home-based medical card and any documents that are informed by the hospital or health clinic to your doctor and pharmacist during the first appointment at the health clinic after discharge from the hospital. Your participation in this study shall end after the appointment. You will not be paid for participating in this study and you will continue with the follow-up treatment as usual. There will be no additional cost incurred to you to participate in this study.

9. What are the benefits of being in this study?
There may or may not be any benefits to you. Information obtained from this study will help improve the management of other patients with the same condition.

10. What are the potential risks and side effects of being in this study?

Participation in this study will not possess any potential risk, as this study does not involve any invasive procedure. The investigator will inform you in a timely manner about any new findings or changes about the study. Where necessary, you may be asked to reconsent to participate.

11. What are my alternatives if I do not participate in this study?

You do not have to participate in this study to get treatment for your disease or condition. You will be treated according to the usual care as per mentioned in point no.6 (group 2).

12. Can the research or my participation be terminated early?

The sponsor may stop the study or your participation at any time. If the study is stopped early for any reason you will be informed.

13. Will my medical information be kept private?

All your information obtained in this study will be kept and handled in a confidential manner, in accordance with applicable laws and/or regulations. When publishing or presenting the study results, your identity will not be revealed without your expressed consent. Individuals involved in this study and in your medical care, or its affiliates and governmental or regulatory authorities may inspect and copy your medical records, where appropriate and necessary.

Data from the study may be archived for the purpose of analysis, but your identity will not be revealed at any time. You will not be informed of the study findings.

14. Who should I call if I have questions?

If you have any questions about the study, please contact the investigator, Mdm Phang Yen Yen at telephone number 01129060439.

If you have any questions about your rights as a participant in this study, please contact: The Secretary, Medical Research & Ethics Committee, Ministry of Health Malaysia, at telephone number 03-3362 8407 / 8205 / 8888.

INFORMED CONSENT FORM

Title of Study: A Randomized Controlled Trial to Compare MedBook Portal and Standard Care in Medication Reconciliation at Transitions of Care from Hospital Discharge to Primary Healthcare Settings

By signing below I confirm the following:

- I have been given oral and written information for the above study and have read and understood the information given.
- I have had sufficient time to consider participation in the study and have had the opportunity to ask questions and all my questions have been answered satisfactorily.
- I understand that my participation is voluntary and I can at anytime free withdraw from the study without giving a reason and this will in no way affect my future treatment. I am not taking part in any other research study at this time. I understand the risks and benefits, and I freely give my informed consent to participate under the conditions stated. I understand that I must follow the investigator's instructions related to my participation in the study.
- I understand that study staff, and governmental or regulatory authorities, have direct access to my medical record in order to make sure that the study is conducted correctly and the data are recorded correctly. All personal details will be treated as STRICTLY CONFIDENTIAL
- I will receive a copy of this subject information/informed consent form signed and dated to bring home.
- I agree/disagree* for my family doctor to be informed of my participation in this study. *(*delete which is not applicable)*

Subject:

Signature: I/C number:

Name: Date:

Investigator conducting informed consent:

Signature: I/C number:

Name: Date:

Impartial witness: *(Required if subject is illiterate and contents of patient information sheet is orally communicated to subject)*

Signature: I/C number:

Name: Date:

Page **4** of **4**

Appendix D: Study Participant Information Sheet and Informed Consent Form in Malay

RISALAH MAKLUMAT PESAKIT DAN BORANG PERSETUJUAN atau KEIZINAN PESAKIT

(untuk subjek dewasa dan penyelidikan intervensi)

1. **Tajuk penyelidikan**:
   Kajian Rawak Kawalan bagi membandingkan 'MedBook Portal' dan Penjagaan Standard dalam semakan rekod ubat sewaktu peralihan rawatan dari Hospital ke Klinik kesihatan

2. **Nama Institusi and nama penyelidik:**
   Phang Yen Yen, Bahagian Perkhidmatan Farmasi, Jabatan Kesihatan Negeri Sarawak
   Professor Dr. Kuan Jew Win, Fakulti Perubatan Dan Sains Kesihatan, Universiti Malaysia Sarawak

2. **Nama penaja**:
   Bahagian Perkhidmatan Farmasi, Jabatan Kesihatan Negeri Sarawak

4. **Pengenalan:**
   Anda telah dijemput untuk menyertai penyelidikan ini kerana anda akan discaj dan dirujuk ke klinik kesihatan kerajaan untuk meneruskan rawatan. Risalah ini menjelaskan hal-hal berkenaan penyelidikan tersebut dengan lebih mendalam dan terperinci. Amat penting anda memahami mengapa penyelidikan ini dilakukan dan apa yang dilakukan dalam penyelidikan ini. Sila ambil masa yang secukupnya untuk membaca dan mempertimbangkan dengan teliti penerangan yang diberi sebelum anda bersetuju untuk menyertai penyelidikan ini. Jika ada sebarang kemusykilan ataupun maklumat lanjut yang anda ingin tahu, anda boleh bertanya dengan mana-mana kakitangan yang terlibat dalam penyelidikan ini. Setelah anda berpuashati
   bahawa anda memahami penyelidikan ini, dan anda berminat untuk turut serta, anda dikehendaki untuk menandatangani Borang Persetujuan, pada muka surat akhir risalah ini.

   Penyertaan anda dalam penyelidikan ini adalah secara sukarela. Anda tidak perlu menyertai penyelidikan ini jika anda tidak mahu. Anda juga mempunyai hak untuk tidak menjawab mana -mana soalan yang anda tidak mahu jawab. Anda juga boleh menarik diri daripada penyelidikan ini pada bila-bila masa. Jika anda menarik diri, segala maklumat yang telah diperolehi sebelum
   anda menarik diri tetap akan digunakan dalam penyelidikan ini. Jika anda tidak mahu menyertai ataupun menarik diri dari penyelidikan ini, tindakan anda tidak akan menjejaskan segala hak dan keistimewaan perkhidmatan perubatan dan kesihatan yang selayaknya anda terima. Penyelidikan ini telah mendapat kelulusan Jawatankuasa Etika dan Penyelidikan Perubatan, Kementerian Kesihatan Malaysia.

5. **Apakah tujuan penyelidikan ini dilakukan?**
   Tujuan penyelidikan ini dilakukan adalah meninjau keberkesanan laman 'MedBook Portal' dalam mengurangkan percanggahan pengubatan sewaktu pesakit dirujuk dari hospital ke klinik
   kesihatan untuk meneruskan rawatan. 'MedBook Portal' ialah sesawang yang membolehkan fasiliti kesihatan KKM berkongsi maklumat pengubatan pesakit. 'MedBook Portal' bertujuan untuk meningkatkan keselamatan pengubatan dan keselamatan pesakit.

   Sejumlah 398 pesakit seperti anda dari empat buah hospital kerajaan akan menyertai penyelidikan ini. Hospital yang terlibat adalah Hospital Umum Sarawak, Hospital Sibu, Hospital Sarikei dan Hospital Miri. Pesakit tersebut akan discaj dari hospital dan dirujuk ke sepuluh buah klinik kesihatan kerajaan. Klinik Kesihatan yang terlibat adalah Klinik Kesihatan Batu Kawa, Klinik Kesihatan Kota Sentosa, Klinik Kesihatan Kota Samarahan, Klinik Kesihatan Jalan Oya, Klinik Kesihatan Lanang, Klinik Kesihatan Sibu

Jaya, Klinik Kesihatan Miri, Klinik Kesihatan Tudan, Klinik Kesihatan Sarikei dan Klinik Kesihatan Bintangor.

Penyelidikan ini akan berlangsung selama 6 bulan dan tempoh pembabitan anda dianggarkan dalam lingkungan 1 minggu sehingga 2 bulan mengikut tarikh temujanji anda di Klinik Kesihatan Kerajaan.

6. Apakah produk penyelidikan yang akan saya terima?

Sekiranya anda bersetuju untuk menyertai penyelidikan ini, anda akan ditentukan secara rawak (secara kebetulan, seperti melambung duit syiling) kepada salah satu kumpulan rawatan yang dinyatakan di bawah. Peluang untuk anda dimasukkan ke mana-mana kumpulan rawatan adalah sama.

Kumpulan 1:
Preskripsi discaj anda akan dimuat naik ke 'MedBook Portal' yang akan digunapakai oleh pegawai penjagaan kesihatan anda di klinik kesihatan kerajaan.
Notis 'MedBook Portal' akan dilekat di muka hadapan 'Home-based Medical Card' anda.
Selepas discaj dari hospital, anda dikehendaki untuk menghadiri temujanji di klinik kesihatan mengikut tarikh yang dijadualkan. Ini merupakan temujanji pertama di klinik kesihatan selepas discaj dari hospital. Sewaktu temujanji ini, anda perlu mengemukakan 'Home-based Medical Card' yang dilekat dengan Notis 'MedBook Portal' bersama sebarang dokumen yang dimaklumkan oleh pihak hospital atau klinik kesihatan, kepada doktor dan pegawai farmasi anda. Doktor anda akan mengkaji kes and berdasarkan preskripsi discaj pada 'MedBook Portal', 'Home-based Medical Card' dan sebarang dokumen yang dikemukakan. Doktor dan pegawai farmasi anda akan menjalankan semakan rekod ubat dengan membandingkan preskripsi discaj pada 'MedBook Portal' dengan preskripsi baru yang disediakan di klinik kesihatan.

Kumpulan 2:
Anda akan diberi penjagaan biasa, di mana tiada 'MedBook Portal' dan tiada Notis 'MedBook Portal' diberi.
Selepas discaj dari hospital, anda dikehendaki untuk menghadiri temujanji di klinik kesihatan mengikut tarikh yang dijadualkan. Ini merupakan temujanji pertama di klinik kesihatan selepas discaj dari hospital. Sewaktu temujanji ini, anda perlu mengemukakan 'Home-based Medical Card' bersama sebarang dokumen yang dimaklumkan oleh pihak hospital atau klinik kesihatan, kepada doktor dan pegawai farmasi anda. Doktor anda akan mengkaji kes and berdasarkan 'Home-based Medical Card' dan sebarang dokumen yang dikemukakan.

Bagi meninjau keberkesanan laman 'MedBook Portal', penyelidik akan membandingkan kedua-dua kumpulan tersebut berkenaan peratusan preskripsi yang ada percanggahan pengubatan melalui aplikasi statistik.

7. Apakah yang terjadi kepada saya sekiranya saya bersetuju untuk menyertai penyelidikan ini?

Anda akan dihubungi oleh penyelidik melalui WhatsApp atau melalui panggilan telefon untuk memberi peringatan mesra mengenakan Tarikh temujanji rawatan susulan di klinik kesihatan kerajaan. Penyelidik akan menghubungi anda melalui nombor telefon 082-473200 atau 01129060439.

8. Apakah tanggungjawab saya sewaktu menyertai penyelidikan ini?

Amat penting anda menjawab kesemua soalan yang ditanyakan oleh kakitangan penyelidikan dengan jujur dan lengkap. Jika keadaan atau kesihatan anda berubah sepanjang penyelidikan ini, anda mesti memberitahu penyelidik anda. Anda perlu mengemukakan 'Home-based Medical Card' bersama sebarang dokumen yang dimaklumkan oleh pihak hospital atau klinik kesihatan, kepada doktor dan pegawai farmasi anda sewaktu menghadiri temujanji pertama di klinik kesihatan selepas discaj dari hospital. Penyertaan anda dalam kajian ini berakhir selepas temujanji tersebut dan anda akan meneruskan rawatan susulan seperti biasa. Anda tidak akan dibayar untuk menyertai kajian ini. Tiada sebarang kos dikenakan pada anda untuk menyertai kajian ini.

9. Apakah manfaatnya saya menyertai kajian ini?

Penyelidikan ini mungkin akan mendatangkan manfaat ataupun langsung tiada memberi apa- apa manfaat kepada anda. Segala maklumat yang diperolehi daripada penyelidikan ini akan dapat membantu dalam penambahbaikan kaedah rawatan atau pengurusan pesakit lain.

10. Apakah risiko dan kesan-kesan sampingan menyertai penyelidikan ini?

Risiko untuk penyertaan penyelidikan ini yang adalah minima dan tidak akan menjejaskan rawatan anda. Dari masa ke semasa, kakitangan penyelidik akan memberitahu anda berkenaan apa-apa penemuan baru atau perubahan berkaitan penyelidikan ini, Jika keadaan memerlukan, anda mungkin akan diminta memberikan persetujuan/keizinan sekali lagi untuk terus menyertai penyelidikan ini.

11. Apakah rawatan alternatif lain sekiranya saya tidak menyertai penyelidikan ini?

Anda tidak perlu menyertai kajian ini untuk mendapatkan rawatan bagi penyakit atau masalah kesihatan anda. Anda akan diberi penjagaan biasa seperti yang diterangkan pada perkara 6 (kumpulan 2).

12. Bolehkah penyelidikan ataupun penyertaan saya ditamatkan lebih awal daripada yang dirancang?

Penaja boleh menamatkan penyelidikan ini ataupun menamatkan penyertaan anda dalam penyelidikan ini pada bila-bila masa. Jika penyelidikan ini dihentikan terlebih awal, di atas sebab-sebab tertentu, anda akan dimaklumkan.

13. Adakah maklumat perubatan saya akan dirahsiakan ?

Segala maklumat anda yang diperolehi dalam penyelidikan ini akan disimpan dan dikendalikan secara sulit, bersesuaian dengan peraturan-peraturan dan/ atau undang-undang yang berkenaan. Sekiranya hasil penyelidikan ini diterbitkan atau dibentangkan kepada orang ramai, identiti anda tidak akan didedahkan tanpa kebenaran anda terlebih dahulu. Pihak- pihak tertentu seperti individu yang terlibat dalam penyelidikan dan rawatan perubatan anda, pihak berkuasa kerajaan atau undang-undang, boleh memeriksa dan membuat salinan laporan perubatan anda jika berkenaan dan diperlukan.

Segala data yang berkaitan dengan penyelidikan ini akan diarkib untuk tujuan analisis, tetapi identiti anda tidak akan didedahkan sama sekali pada bila-bila masa. Anda tidak akan dimaklumkan hasil kajian.

14. Siapakah yang perlu saya hubungi sekiranya saya mempunyai sebarang pertanyaan?

Anda boleh menghubungi penyelidik ini, Pn. Phang Yen Yen pada sambungan telefon 01129060439 sekiranya anda mempunyai sebarang pertanyaan mengenai penyelidikan ini.

Jika anda mempunyai sebarang pertanyaan berkaitan dengan hak-hak anda sebagai pesakit dalam penyelidikan ini, sila hubungi: Setiausaha, Jawatankuasa Etika & Penyelidikan Perubatan, Kementerian Kesihatan Malaysia, melalui talian telefon 03-3362 8407/ 8205 / 8888.

BORANG PERSETUJUAN/ KEIZINAN PESAKIT

Tajuk Penyelidikan: Kajian Rawak Kawalan bagi membandingkan 'MedBook Portal' dan Penjagaan Standard dalam semakan rekod ubat sewaktu peralihan rawatan dari Hospital ke Klinik kesihatan

Dengan menandatangani di bawah, saya mengesahkan bahawa :

- Saya telah diberi maklumat tentang penyelidikan di atas secara lisan dan bertulis and saya telah membaca dan memahami segala maklumat yang diberikan dalam risalah ini.
- Saya telah diberikan masa yang secukupnya untuk mempertimbangkan penyertaan saya dalam penyelidikan ini dan telah diberi peluang untuk bertanyakan soalan dan semua persoalan saya telah dijawab dengan sempurna dan memuaskan.
- Saya juga faham bahawa penyertaan saya adalah secara sukarela dan pada bila-bila masa saya bebas menarik diri daripada penyelidikan ini tanpa harus memberi sebarang alasan dan ianya sama sekali tidak akan menjejaskan rawatan perubatan saya pada masa akan datang. Saya tidak mengambil bahagian dalam mana-mana penyelidikan lain pada masa ini. Saya juga memahami tentang risiko dan manfaat penyelidikan ini dan saya secara sukarela memberi persetujuan untuk menyertai penyelidikan ini di bawah syarat-syarat yang telah dinyatakan di atas. Saya faham saya harus mematuhi nasihat dan arahan yang berkaitan dengan penyertaan saya dalam penyelidikan ini daripada penyelidik.
- Saya faham bahawa kakitangan penyelidikan, dan pihak berkuasa kerajaan atau undang-undang, mempunyai akses langsung dan boleh menyemak laporan perubatan saya bagi memastikan penyelidikan ini dijalankan dengan betul dan data direkodkan dengan betul. Segala maklumat dan data peribadi akan dianggap sebagai SULIT.
- Saya akan menerima satu salinan 'Risalah Maklumat Pesakit dan Borang Persetujuan atau Keizinan Pesakit' yang telah lengkap dengan tarikh dan tandatangan untuk dibawa pulang ke rumah.
- Saya bersetuju/ tidak bersetuju* untuk doktor yang biasa merawat saya diberitahu tentang penyertaan saya dalam penyelidikan ini. *(*Potong mana yang tidak berkenaan)*

Subjek :

Tandatangan: Nombor K/P:

Nama: Tarikh :

Penyelidik yang mengendalikan proses menandatangani borang keizinan:

Tandatangan: Nombor K/P:

Nama: Tarikh :

Saksi tidak-berpihak/adil: *(Diperlukan; jika subjek adalah buta huruf dan kandungan risalah maklumat pesakit disampaikan secara lisan kepada subjek)*

Tandatangan: Nombor K/P:

Nama: Tarikh :
