# Supplementary material for: Effectiveness of digital platform in reducing unintentional medication discrepancies at transition of care from hospital discharge to primary healthcare settings: a randomised controlled trial
Source: BMC Prim Care. 2025 Jul 2;26:206. doi: 10.1186/s12875-025-02904-z (PMC12219133; doi:10.1186/s12875-025-02904-z)
Supplement: Supplementary file 2 — Supplementary Material 2 [file 12875_2025_2904_MOESM2_ESM.pdf]

Effectiveness of Digital Platform in Reducing Unintentional Medication Discrepancies at Transition of Care from Hospital Discharge to Primary Healthcare Settings: A Randomised Controlled Trial

Yen Yen Phang[1,2*], Jew Win Kuan[1], Ai Ling Oh[3], Chuo Yew Ting[2], Nor Anizah Osman[2], Stephen Moses[2]

[1]Faculty of Medicine and Health Sciences, Universiti Malaysia Sarawak (UNIMAS), Kota Samarahan, Malaysia.

[2]Pharmacy Service Division, Sarawak State Health Department, Ministry of Health, Kuching, Malaysia.

[3]Pharmacy Department, Sarawak General Hospital, Ministry of Health, Kuching, Malaysia.

*Correspondence:

Yen Yen Phang

phangyenyen@gmail.com

SUPPLEMENTARY INFORMATION – ADDITIONAL FILE

1.1 MedBook Portal

MedBook portal (https://aplikasi.jknsarawak.moh.gov.my/medbook) is a digital platform that enable sharing of patient medication records among healthcare facilities under Ministry of Health. The Sarawak State Health Department owns and manages this portal, with all information stored on their server. The portal is accessible via both smartphone and computers, with the smartphone version's design depicted in Figure A and the computer version's design shown in Figure B. Healthcare providers, including doctors and pharmacists in the primary health clinics (PHCs), are granted access to this portal using their Identification Number and a password they created.

During the discharge process, hospital study pharmacists upload the discharge prescription (Figure C) to MedBook Portal for both the Standard Care group and the MedBook Portal group. When uploading the discharge prescription, the hospital study pharmacist selects the 'intervention' radio button for patients in the MedBook Portal group. This setting allowed PHC doctors to view prescription only for patients in the MedBook Portal group, while PHC pharmacists can view prescriptions for all patients. This differentiation is based on user roles within the MedBook Portal, facilitating study pharmacists' comparison of discharge prescriptions and first prescriptions at first PHC visit to identify unintentional medication discrepancies in both groups. Figure D showed the difference in the 'intervention' radio button setting of MedBook Portal between the Standard Care Group and the MedBook Portal Group.

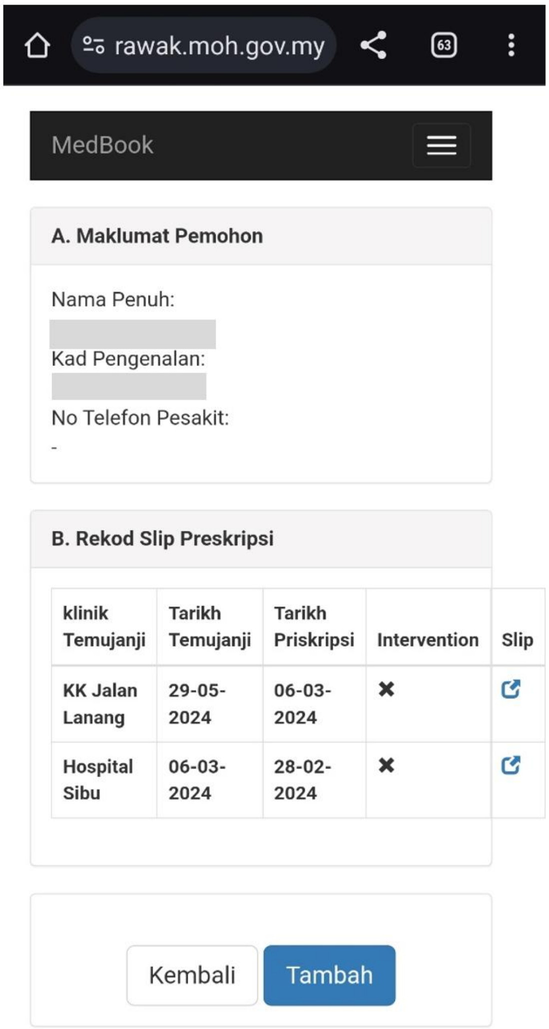

Figure A: MedBook Portal in Smartphone Version

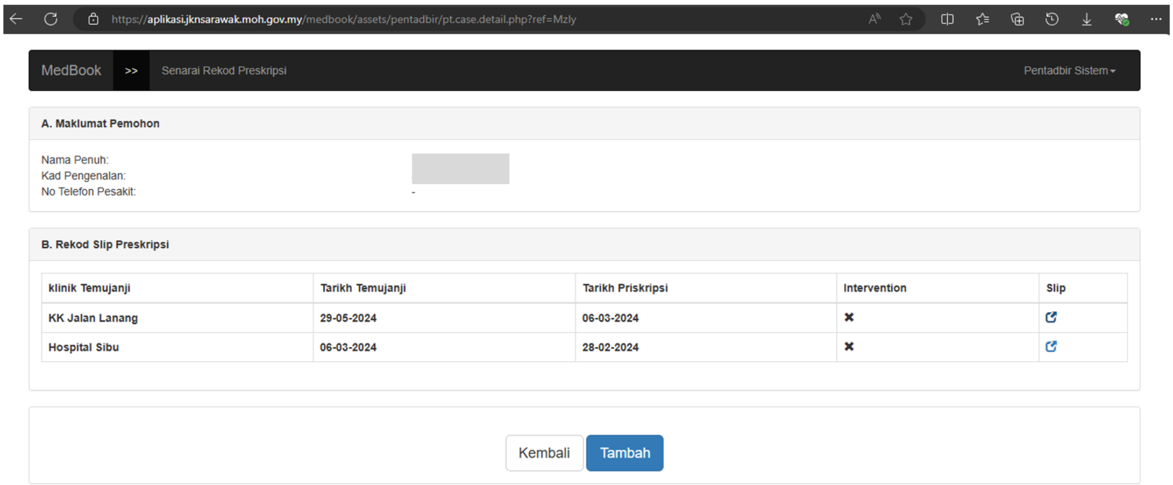

Figure B: MedBook Portal in Computer Version

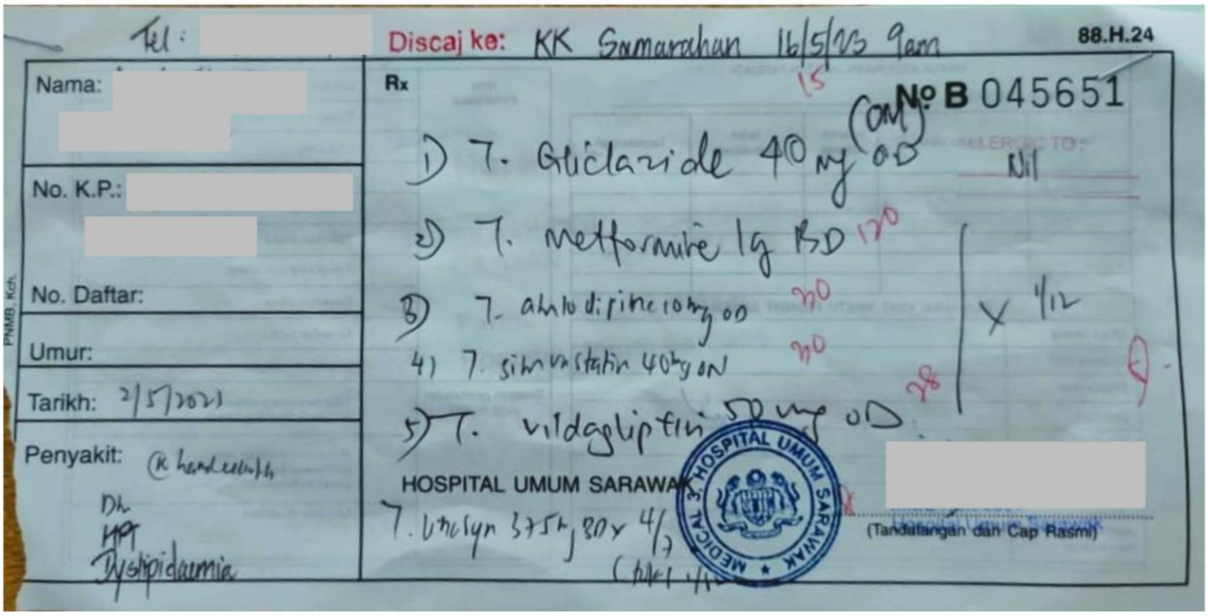

Figure C: Example of Discharge Prescription Uploaded in Medbook Portal

Subject in Standard Care Group

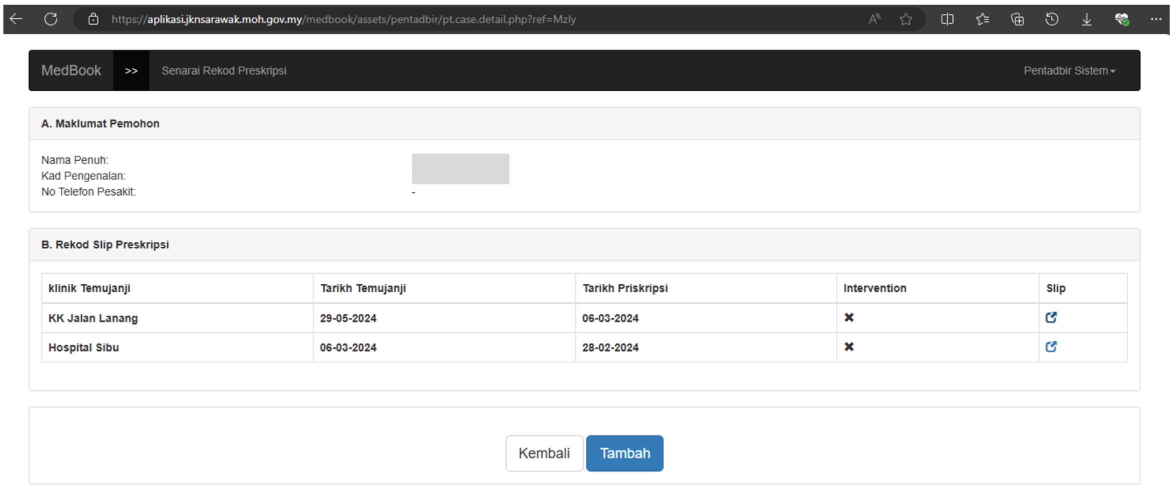

Subject in MedBook Portal Group

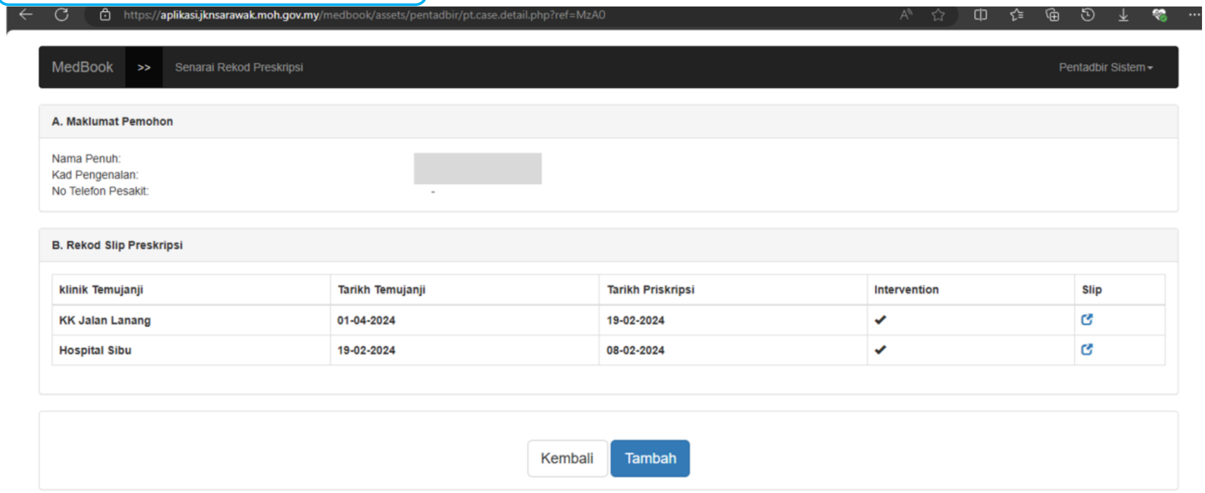

Figure D: Difference in the 'Intervention' Radio Button Setting of Medbook Portal between the Standard Care Group and the MedBook Portal Group

1.2 MedBook Portal Notice

A MedBook Portal Notice was affixed to front of each patient’s home-based medical card, exclusively for those in the MedBook Portal group. This allowed PHC doctors and PHC pharmacists to easily identify patients recruited into the MedBook Portal group. The layout of the MedBook Portal Notice is illustrated in Figure E.

IMPORTANT

Dear Prescriber,

Please log in to MedBook Portal **https://aplikasi.jknsarawak.moh.gov.my/medbook** using Myworkspace ID & Password to retrieve patient’s latest medication list.

Thank you.

Regards,
MedBook Portal Team

Scan me!

Figure E: MedBook Portal Notice

At Hospital

Medication History Assessment Form (CP1)

T. Metoprolol 50mg BD
T. Gliclazide MR 60mg OD
T. Atorvastatin 20mg ON
T. Pantoprazole 40mg OD
T. Aspirin 75mg OD

Inpatient Medication Chart

T. Losartan 100mg OD
T. Gliclazide MR 60mg OD
T. Atorvastatin 40mg ON
IV. Pantoprazole 40mg OD
T. Neurobion l/l OD

Reconciliation at Hospital Discharge

Discharge Prescription

T. Losartan 50mg BD
T. Gliclazide 60mg OD
T. Atorvastatin 40mg ON
T. Cardiprin 100mg OD
T. Neurobion l/l OD
T. Mecobalamin 500mcg TDS

Medication Discrepancy Identified		
Drug	*Type*	*Justification*
T. Losartan 50mg BD	*Unintentional Discrepant Dose*	*Should be 100mg*
T. Losartan 50mg BD	*Unintentional Discrepant Frequency*	*Should be OD*
T. Gliclazide 60mg OD	*Unintentional Others*	*Should be Gliclazide MR*
T. Cardiprin 100mg OD	*Intentional Drug Addition*	*From Aspirin to Cardiprin*
T. Mecobalamin 500mcg TDS	*Unintentional Therapeutic Duplication*	*Neurobion was prescribed*
T. Metoprolol 50mg BD	*Intentional Drug Omission*	*No longer indicated*
T. Pantoprazole 40mg OD	*Unintentional Drug Omission*	*Should be continued*
T. Aspirin 75mg OD	*Intentional Drug Omission*	*No longer indicated*

RECONCILIED Discharge Prescription

T. Losartan 100mg OD
T. Gliclazide MR 60mg OD
T. Atorvastatin 40mg ON
T. Cardiprin 100mg OD
T. Neurobion l/l OD
T. Pantoprazole 40mg OD

Reconciliation at Primary Health Clinic

At Primary Health Clinic

First Prescription during the First Primary Health Clinic Visit After Hospital Discharge

T. Metoprolol 100mg BD
T. Gliclazide MR 90mg OD
T. Atorvastatin 20mg ON
T. Pantoprazole 40mg OD
T. Aspirin 75mg OD
T. Paracetamol 1g PRN

Medication Discrepancy Identified		
Drug	*Type*	*Justification*
T. Metoprolol 100mg BD	*Unintentional Drug Addition*	*No longer indicated*
T. Gliclazide MR 90mg OD	*Intentional Discrepant Dose*	*Increased dose*
T. Atorvastatin 20mg ON	*Unintentional Discrepant Dose*	*Should be 40mg*
T. Aspirin 75mg OD	*Unintentional Drug Addition*	*No longer indicated*
T. Paracetamol 1g PRN	*Intentional Drug Addition*	*Indicated*
T. Losartan 100mg OD	*Unintentional Drug Omission*	*Should be continued*
T. Cardiprin 100mg OD	*Unintentional Drug Omission*	*Change from Aspirin to Cardiprin*
T. Neurobion l/l OD	*Unintentional Drug Omission*	*Should be continued*

Figure F: Illustration of Medication Discrepancies Detected during Medication Reconciliation at Hospital Discharge and First PHC Visit

Table A: Comparison of Baseline Characteristics between the Groups (n = 398)

Variables	Overall (n=398)	MedBook Portal Group (n=199)	Standard Care Group (n=199)	*p* value
**Study sites**, n (%)				
Sarawak General Hospital	176 (44.2)	88 (44.2)	88 (44.2)	0.997[a]
Sarikei Hospital	100 (25.1)	50 (25.1)	50 (25.1)	
Sibu Hospital	95 (23.9)	48 (24.1)	47 (23.6)	
Miri Hospital	27 (6.8)	13 (6.6)	14 (7.1)	
**Age,** mean years (SD)	63.2 (±13.9)	64.1 (±13.6)	62.3 (±14.1)	0.196[b]
**Gender**, n (%)				
Male	203 (51.0)	104 (52.3)	99 (49.7)	0.616[a]
Female	195 (49.0)	95 (47.7)	100 (50.3)	
**Ethnicity**, n (%)				
Sarawak Native	176 (44.2)	93 (46.7)	83 (41.7)	0.336[a]
Chinese	130 (32.7)	60 (30.2)	70 (35.2)	
Malay	90 (22.6)	44 (22.1)	46 (23.1)	
Indian	2 (0.5)	2 (1.0)	0 (0.0)	
**Number of comorbidities,** median (IQR)	3 (2-4)	3 (3-4)	3 (2-4)	0.076[c]
**Number of medications in Discharge Prescription,** mean (SD)	6.94 (±2.88)	7.48 (±2.84)	6.40 (±2.82)	**<0.001[b]**

[a]Pearson's chi-squared test
[b]Independent t test
[c]Mann-Whitney U test
IQR: Interquartile Range
SD: Standard Deviation
